# Optimal prey for red fox cubs—An example of dual optimizing foraging strategy in foxes from a dynamic wetland habitat

**DOI:** 10.1002/ece3.10033

**Published:** 2023-04-20

**Authors:** József Lanszki, Zsolt Bende, Nikolett Nagyapáti, Zsófia Lanszki, Péter Pongrácz

**Affiliations:** ^1^ Fish and Conservation Ecology Research Group Balaton Limnological Research Institute Tihany Hungary; ^2^ Balaton Uplands National Park Directorate Csopak Hungary; ^3^ Duna‐Ipoly National Park Directorate Budapest Hungary; ^4^ Institute of Biology University of Pécs Pécs Hungary; ^5^ Department of Ethology ELTE Eötvös Loránd University Budapest Hungary

**Keywords:** cub provisioning, energy content, marshland, optimal foraging, predation, prey size

## Abstract

The red fox (*Vulpes vulpes*) is the most abundant mesopredator in the Central European region. Detailed knowledge about their feeding behavior is important both from ecological and wildlife management reasons. Food choices of foxes are poorly predictable in high‐biodiversity marshlands. The main aim of our study was to sample parallel the main food‐type abundances in the study area and analyze the diet of fox cubs and cohabiting adults across 3 years during the period of maternal dependence of the cubs. According to the optimal foraging theory, we predicted that the cubs' diet would show higher energy content, would be more varied, and the individual prey species fed to the young would be larger. We analyzed the composition of adult fox and cub fecal samples collected separately around dens in a marshland of western Hungary, May 2014, 2017 and 2020, when the abundance values of main food sources differed. Rodents and waterfowl dominated the diet, but their relative occurrence in the samples showed yearly variations. We found that vixens follow a dual optimizing foraging strategy regarding their provisioning of the cubs and their own diet. Adult foxes optimized their diet according to the actual yearly abundances of their main food sources. Additionally, they preferred prey items that can be consumed at the site of capture (large carrion and small individual prey items). Cubs on the other hand were provisioned with optimal high‐energy food, even if those in question became less abundant in that year. Vixens mostly fed to their young either larger rodents and waterfowl, or multiple small rodents at a time—these type of prey are both optimal for transportation as a single load. Providing optimal prey at an early age in a changing environment may contribute to the ecological success of the red fox.

## Graphical abstract

The diet of red fox cubs was evaluated against the naturally occurring prey availability. Age group‐ and year‐dependent dietary patterns suggest the effect of optimization in maternal investment. The cubs are provided with varied, high‐energy content prey items. The mother selectively provides large‐sized rodents and birds for the cubs. Maternal foraging pattern found in a wetland habitat can be regarded as optimal.

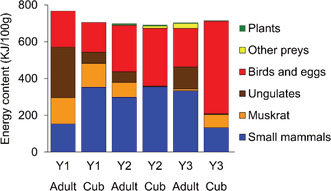

## 1 INTRODUCTION

The optimal foraging theory (OFT), a behavioral ecology model, postulates that foragers select food items by finding a compromise between costs and benefits (e.g., finding the net energy gain optimum where travel distance between two food patches adds to the cost and energy intake in the next visited food patch represents the benefit, Cowie, 1977) to maximize their net rates of energy intake, where optimal net energy intake has a direct association with fitness (Krebs, 1978; MacArthur & Pianka, 1966; Pyke et al., 1977; Stephens & Krebs, 1986). Based on the OFT, it is also expected that a generalist forager will increase dietary diversity in response to a decrease in prey or the availability of its preferred (primary) prey (Begg et al., 2003; MacArthur & Pianka, 1966; Perry & Pianka, 1997).

According to the parental investment theory (PIT), one key component is rearing investment, which is, the time and energy expenditure connected directly to the raising of offspring (Geffen et al., 1996; Geffen & MacDonald, 1992; Moehlman, 1986; Trivers, 1972; Vergara, 2000). In many species, parental investment includes feeding the offspring that are not yet able to obtain food on their own. Beyond fulfilling their energetic needs, juvenile terrestrial carnivores need to recognize and learn about prey species in the environment based on taste and smell at a young age (Blandford, 1987; Thornton & Raihani, 2008). Multiple factors determine how effective predatory behavior arises. One of these factors is the role of the mother, for example in cats, kittens are stimulated to catch prey by bringing living prey to them (Ewer, 1969). Presenting a variety of food items to the offspring by the parents influences their food choice after becoming independent (Altbäcker et al., 1995; Blandford, 1987; Thornton & Raihani, 2008). It is expected, however, that even in the case of a variable (quantity and composition) food source, the parent will provide the offspring with the optimal food that has the highest net energy content.

The prey abundance‐ and composition‐dependent feeding (functional) response (or prey selection), as a behavioral response (Holling, 1959; Jeschke et al., 2002; Murdoch, 1969; Randa et al., 2009) has been less explored in carnivores during the early stages of progeny rearing, when the offspring are still dependent on the parent(s). Terrestrial carnivores, such as the species of Felidae, Mustelidae, and Canidae, have to return to their still‐dependent young with captured prey to feed them. This scenario is very similar to the well‐studied cases of birds where the association between optimal foraging and optimal parental investment strategies was thoroughly modeled and analyzed (e.g., Fargallo et al., 2020). In a nutshell, parents who have to retrieve prey to a central location (i.e., to the nest/den where their young are), have to find the optimal solution that minimizes the cumulative travel distance and provides the most nutritious prey composition to the offspring (Kacelnik & Houston, 1984). Additional restrictive factors emerge in the form of the capturability of the various prey types, the transport cost, and the potential exposure of the parents to dangers (such as predators, including humans; Brown, 1999).

The red fox (*Vulpes vulpes*) is an opportunistic and generalist predator that responds functionally to seasonal and geographic fluctuations of prey and plant resources, resulting in a varied diet composition across their range (e.g., Europe: Díaz‐Ruiz et al., 2013; Lloyd, 1980; Soe et al., 2017; North America: Sargeant et al., 1984; Australia: Saunders et al., 2010; globally: Castañeda et al., 2022). Due to its population size, the red fox is one of the most significant mesopredator globally (Doherty et al., 2016; Lloyd, 1980; Soe et al., 2017).

Due to its ubiquitous presence around the world's cold and temperate zone habitats and cities (Baker & Harris, 2004; Doherty et al., 2016; Doncaster et al., 1990; Lloyd, 1980), the red fox can be an ideal model species for the study of OFT and optimal parental provisioning behavior in predatory mammals. Red foxes are facultative polygamous canids (Baker et al., 2004; Macdonald, 1979; Moehlman, 1979; Zabel & Taggart, 1989). Their mating system (monogamy vs. polygamy; Macdonald, 1979; Zabel & Taggart, 1989) is strongly influenced by environmental factors, for example, the abundance of prey and fox density. Similarly, in some instances the presence of barren (subordinate) vixens were recorded, who took the role of “helpers” by provisioning the cubs of the higher‐ranking female (Baker et al., 2004; Macdonald, 1979). In Central Europe cubs are mainly born in April, leaving the den for the first time at 4 weeks of age (Bekoff et al., 1981; Lloyd, 1980). At 3 weeks of age, the consumption of solid food begins and at 4–5 weeks of age weaning is completed (Kolb & Hewson, 1980). Instead of the regurgitated food typical in the early period of maternal provisioning, the mother provides the cubs with more or less intact prey items toward the end of cub‐rearing (Nygaard, 1990). Due to their typically small‐to‐medium sized, non‐dangerous prey items, foxes do not rely on cooperative hunting, thus the young disperse early (depending on habitat, from 6 months of age; e.g., Harris & Trewhella, 1988; Lloyd, 1980), and become reproductively active quickly (10‐months‐of‐age; Baker et al., 1998; Bekoff et al., 1981; Kolb & Hewson, 1980; Macdonald, 1983).

In comparative studies, the food composition of recently weaned cubs and adult foxes was different (Artois & Gall, 1988; Weber, 1996), but the diet of young and adult foxes was similar once the young animals became independent of parental provisioning (Catling, 1988; Cavallini & Volpi, 1996; Balestrieri et al., 2011; Kolb & Hewson, 1980; exceptions, Kidawa & Kowalczyk, 2011; Lever, 1959). These results indicate that fox mothers may actively select prey items that could be more advantageous from the aspect of parental provisioning. The prey brought back to the cubs by the parents was usually larger than the typical prey size of the adult red foxes living in the same area (Lindström, 1994; Lovari & Parigi, 1995; Weber, 1996).

The differences in habitat conditions (originating either from effects of locality or temporal changes), for example, in prey abundance, are usually well reflected in the diet composition of vertebrate predators and community dynamics (Balestrieri et al., 2011; Díaz‐Ruiz et al., 2013; Jędrzejewski & Jędrzejewska, 1992; Kjellander & Nordström, 2003; Panzacchi et al., 2008; Soe et al., 2017; but see methodological limitations: Reynolds & Aebischer, 1991). To get a clear picture of the diet of a carnivore, indirect methods are often used such as assessing particular traces of the consumed prey instead of observing the actual event of predation. Prey species detected in the stomach or feces of carnivores can provide important insight into feeding habits (Castañeda et al., 2022; Machovsky‐Capuska et al., 2016; Saunders et al., 1993; Soe et al., 2017) and opportunity for quantitative analysis of the food consumed (Reynolds & Aebischer, 1991), in contrast to stable isotopic and DNA barcoding methods (e.g. De Barba et al., 2014; Newsome et al., 2007) or direct observation (e.g., Lloyd, 1980; Macdonald, 1977), which are novel from another aspects.

Compared to drylands and anthropogenic ecosystems (Baker et al., 2006; Díaz‐Ruiz et al., 2013; Soe et al., 2017), marshes consist of specific natural transitional areas of wetlands and terrestrial habitats (Mitsch & Gosselink, 1986), where little is known about the behavioral ecological background of prey choice of foxes (Goldyn et al., 2003; Lanszki, 2005; Leckie et al., 1998; Reynolds, 1979). Marshlands are ecologically optimal areas of high productivity and biodiversity and host unique and, contrary to drylands, more stable prey communities (Dell'Arte et al., 2007; Scott et al., 2008; Verboom et al., 2006). Although predation on nesting waterfowl or other species associated with aquatic habitats may be significant in marshlands (e.g., Purger et al., 2023; Sargeant et al., 1984), there is an almost complete lack of assessment of OFT‐based prey choice of adult red foxes regarding their self‐sustenance and parallel provisioning of dependent cubs (Weber, 1996).

Based on the OFT and earlier findings in red foxes (Lindström, 1994; Weber, 1996), we hypothesize that prey choice of adults will depend on the relative food availability at a given location and year. Our first prediction is that adult fox food preferences in a given year will depend on the general food abundance. Our second prediction is that prey consumed at the capture site (feeding preference of adult foxes) and prey brought back to the den for cubs (food composition of cubs) should be different according to the predictions of optimized parental provisioning models (i.e., cubs should be steadily provided with high‐energy content prey; prey brought back from larger distances should be profitable based on the energy content/cost of transportation). We also expected that the need for optimal provisioning for their dependent cubs would have a stronger influence on maternal prey choice than the fluctuating yearly prey abundance. Therefore, we predicted (third prediction, based on the interaction of factors) that the differences between adult feeding patterns and the composition of the cubs' diet will be maintained across the years. To explore the quality of parental investment via age‐ and source‐dependent dietary differences, we examined the food selection of adult red foxes living in natural conditions. We simultaneously collected fecal samples of the adults and fox cubs as well from different years during the cubs' dependency phase, when they were fed with prey brought back by their mothers. From the qualitative analysis of fecal samples (composition, prey size and habitat/zonation, preferences, energy content), we assessed how flexibly the mother optimized the prey selection for the cubs. We expected that prey brought back to cubs would be different in composition (i.e., predicting higher energy content; and regarding the marshland habitat, higher proportion of aquatic prey), more varied (thus probably enhancing the effect of gaining experience with a wider assortment of prey), and the individual prey items would be larger, depending on the abundance of available food sources.

## 2 MATERIALS AND METHODS

### 2.1 Study area

**FIGURE 1:**
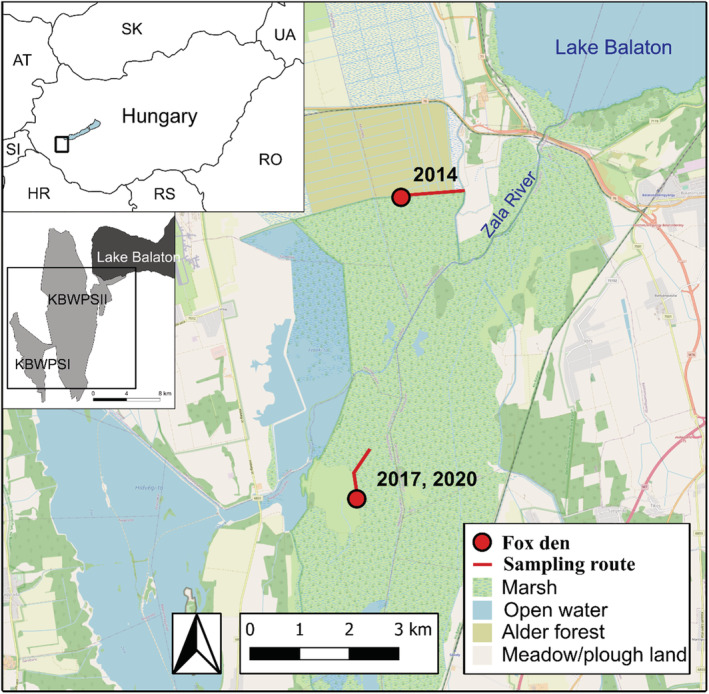
Map of the study area. The year is indicated at the den sites when the particular site was used for sampling.

The study area is situated in the western part of Hungary, in the Kis‐Balaton area of Balaton Uplands National Park (Figure 1). Our investigations were performed within the strictly protected 54 km^2^ Kis‐Balaton marshland (center: 46.63723° N, 17.20938° E). Kis‐Balaton has been a Natura 2000 site (SPA—Special Protection Areas and PSCI—Proposed Sites for Community Importance) and a Ramsar site since 1979. The main habitat types in the marshland are the following: open water (10%), swamp vegetation (74%), grasslands (3%), wooded habitats (12%), built environment (gravel roads, built structures; <1%) (Balaton Uplands National Park's database). The dominant species of the plant communities in the swamp are common reed (*Phragmites australis*), common bulrush (*Typha latifolia*), lesser pond‐sedge (*Carex acutiformis*), at higher spatial levels, patches of groves are formed by willows (*Salix alba* and *S. cinerea*), poplars (*Populus* sp.), and common alder (*Alnus glutinosa*).

The shores of the embankments reserved for water‐level regulation are suitable for the foxes to dig their dens. These linear infrastructures offer safe passage between habitat patches at a higher spatial level (e.g., islands) for foxes in all seasons. At the same time, these pathways ensure the collection of scat samples for dietary studies. Human activity within the study area is very low; mostly involving conservation management and water protection (Lanszki et al., 2020).

The study area is situated in the continental climatic region, with some Mediterranean features. During the study periods (2014, 2017, and 2020) in spring (from March to May), no significant differences were found among the years in mean (±SE) monthly temperature (11.6 ± 0.9°C; ANOVA, *F*_2,6_ = 0.05, *p* = .945) and mean monthly amount of precipitation (30.9 ± 5.7 mm, *F*_2,6_ = 1.76, *p* = .250; data source: Hungarian Meteorological Service).

There is a large number of waterfowl nests at Kis‐Balaton, and the area is an important wintering and migration area for birds and a favorable habitat for amphibians and invertebrates (Lanszki et al., 2020). Red deer (*Cervus elaphus*) and wild boar (*Sus scrofa*) populations are abundant, while roe deer (*Capreous capreolus*) and European brown hare (*Lepus europaeus*) are rare species here.

### 2.2 Sample collection and study species

For the assessment of the diet composition and feeding habits of the red fox, we collected 265 intact scat (fecal) samples in 3 years (Table 1), Y1 (year 1–2014), Y2 (year 2–2017), Y3 (year 3–2020). Scat samples of cubs and an additional 14 partly consumed prey remains were collected in the surroundings of inhabited dens with actual ongoing cub‐rearing, within a distance of 3–5 m from one den with cubs per year. Scat samples from adult foxes were collected weekly once, by walking slowly on standard routes designated for carnivore monitoring (Figure 1), and all samples stored frozen until analysis. Both types of scats were collected on several occasions (on average once a week) during May. The standard monitoring route around the dens was 1.7 km in Y1 and 1.3 km in Y2 and Y3. From June, as the cubs began to switch to independent foraging, the dens were abandoned, and scat sample collection has been terminated.

**TABLE 1:** Percentage relative frequency of occurrence (RFO), frequency of occurrence (FO) and estimated biomass (BC) of main food categories in fecal samples of adult and cub red foxes (*Vulpes vulpes*) in the Kis‐Balaton marshland (Hungary).

Food categories	Y1						Y2						Y3					
	Adult (*n* = 33)			Cub (*n* = 42)			Adult (*n* = 32)			Cub (*n* = 63)			Adult (*n* = 47)			Cub (*n* = 48)		
	RFO	FO	BC	RFO	FO	BC	RFO	FO	BC	RFO	FO	BC	RFO	FO	BC	RFO	FO	BC
Small rodents	26.5	66.7	22.1	49.4	85.7	53.1	35.4	78.1	44.8	26.8	69.8	53.4	40.5	68.1	50.2	20.7	35.4	19.9
Insectivores	2.4	6.1	1.2							0.6	1.6	+						
Muskrat	4.8	12.1	19.9	8.9	16.7	18.0	1.3	3.1	11.1				1.3	2.1	1.3	4.9	8.3	10.0
Ungulates	12.0	30.3	30.4	6.3	11.9	6.9	6.3	15.6	6.6	1.2	3.2	0.6	8.9	12.8	13.4	1.2	2.1	0.6
Carnivores										0.6	1.6	1.7						
Birds	24.1	48.5	25.2	29.1	50.0	21.8	25.3	62.5	34.0	41.5	82.5	38.7	26.6	42.6	27.6	62.2	93.8	64.9
Bird eggs	10.8	27.3	1.2				3.8	9.4	0.1	7.3	19.0	3.5	2.5	4.3	0.6	6.1	10.4	3.1
Reptiles	2.4	6.1	+				2.5	6.3	+	0.6	1.6	+				1.2	2.1	0.1
Amphibians	1.2	3.0	+				1.3	3.1	+	0.6	1.6	+						
Fish							3.8	9.4	0.5	0.6	1.6	+	5.1	8.5	5.2	1.2	2.1	+
Invertebrates	13.3	36.4	+	5.1	9.5	+	12.7	21.9	+	12.8	30.2	0.1	8.9	14.9	0.1			
Plants	2.4	6.1	+	1.3	2.4	0.2	7.6	18.8	2.8	7.3	17.5	2.0	6.3	10.6	1.6	2.4	4.2	1.3
Number of items (i)	83			79			79			164			79			82		
*i*/*n*	2.52			1.88			2.47			2.60			1.68			1.71		
B	5.67		4.09	2.91		2.73	4.56		2.99	3.69		2.29	3.88		2.86	2.29		2.12
B_A_	0.17		0.05	0.10		0.06	0.20		0.12	0.13		0.07	0.11		0.05	0.11		0.09
Shannon diversity	0.56		0.67	0.63		0.64	0.58		0.66	0.62		0.66	0.60		0.64	0.55		0.55
Prey species richness	23			15			21			23			17			15		
Mean energy (KJ/100 g)			723.5			688.7			682.7			691.3			701.8			709.1
±SE			13.7			5.5			17.5			7.9			13.3			11.5

*Note*: Fecal samples were collected during the cub‐rearing period in Y1 (first year—May 2014), Y2 (second year—May 2017) and Y3 (third year—May 2020). *i*/*n*—number of food items per sample. B—trophic niche breadth value (Levins, 1968). B_A_—standardized trophic niche breadth value (Hurlbert, 1978). ±SE—standard error value to mean energy content data. +—biomass <0.05%. Empty cells in the diet composition panel mean that the given food type was not detected.

The same fox den was studied in Y2 and Y3 and a distant one (8.9 km, measured on embankments) in Y1. We opted for using the scat samples collected 3 years apart because this likely ensured that the fox mothers were not the same individuals even at the same den site. Based on numerous published results (e.g., Harris, 1979; Hartová‐Nentvichová et al., 2010; Hradsky et al., 2019; Phillips, 1970) from a wide range of habitats, the ratio of foxes older than 4 years in the population is low to negligible.

Fox scat samples were distinguished from other sympatric carnivores based on position, odor, size, and shape characteristics (Castillo et al., 2011; Jędrzejewska & Jędrzejewski, 1998; Lanszki, 2005; Macdonald, 1980). Scats of adult foxes were easily distinguishable from those of cubs based on their larger size. The mean (±SE) dry weight of washed and dried scat samples of cubs were only one‐third size of samples from adult foxes (0.61 ± 0.04 g vs. 1.77 ± 0.14 g; Mann–Whitney *U*‐test, *U* = 3719.5, *n*_1_ = 153, n_2_ = 112, *p* < .0001). Based on camera trapping (Y1 and Y3) and occasional observations (Y2), the vixens raised the cubs alone, without helpers (Macdonald, 1983). The number of cubs was at least three in Y1 and minimum of two in Y2 and Y3; their age ranged between 4 and 8 weeks during the sample collection.

The relative abundance of potentially reproducing red foxes (individuals per km survey route) was calculated on the basis of den number (inhabited fox den × 2) by surveys performed in March (and afterward checked dens each month in spring), 2010–2020. Thus, the estimated mean (±SE) red fox abundance index was 0.57 ± 0.06 fox/km per year (lower quartile: 0.40 fox/km, upper quartile: 0.66 fox/km; J. Lanszki, unpublished data). The yearly values in the selected 3 years were medium high, 0.63, 0.42, and 0.63 fox/km, respectively.

For calculating the food preference indices of the foxes, we used the small mammal abundance data of the three selected years. We calculated the abundance (MNA, or minimum number alive/100 trap nights) of the small mammal (rodents and insectivores) population in the Kis‐Balaton marshland. During these surveys, we followed the four‐night trapping by mark–recapture technique (Krebs, 1989). Live trapping was conducted yearly in May 2010–2020. The mean (±SE) small mammal abundance (MNA) was 7.0 ± 1.3 individuals/100 trap nights (lower quartile: 3.7 individuals/100 trap nights, upper quartile: 9.1 individuals/100 trap nights; J. Lanszki, unpublished data). According to these abundance data (non‐normal data distribution, Kruskal–Wallis median test, *χ* ^2^ = 25.23, *p* = .002), the abundance of small mammals significantly differed across the years (Table S1), it was the highest in Y1 (15.5 individuals/100 trap nights), medium in Y2 (4.0 individuals/100 trap nights), and the lowest in Y3 (2.8 individuals/ 100 trap nights).

Among the birds in the area, the population density of waterfowl averaged 10.5 ± 1.3 individuals/km^2^, based on standard censuses done by direct binocular and spotting scope counting in April and May 2010–2020 (lower quartile: 9.6 individuals/km^2^, upper quartile: 12.6 individuals/km^2^; source of synchronous bird censuses: Balaton Uplands National Park Directorate's database). Based on the whole (10‐year) monitoring period, the abundance of waterfowl was intermediate (10.2 and 11.2 individuals/km^2^) in Y1 and Y2 and low (4.4 individuals/km^2^) in Y3. High predation rates, caused mainly by foxes, in artificial nest predation tests were found at nest sites of waterfowl (13%–28%, J. Lanszki, unpublished monitoring data).

All studies within the strictly protected area, including live trapping of small mammals, were permitted by the Balaton Uplands National Park Directorate and the local, competent authority (VAV/KTF/1258‐8/2015, ZA/KTF/01115‐7/2017, ZA/KTF/02051‐8/2020). We followed all applicable international, national, and/or institutional guidelines for the care and use of animals.

### 2.3 Fecal sample analysis

To ensure that the collected fecal samples originated from foxes, due to the lack of genetic species identification (e.g., Baines et al., 2013), we rechecked the questionable samples during the laboratory preparation procedure, for example, based on typical odor (Lanszki et al., 2020). Additionally, carnivore hairs collected from fecal samples were morphologically identified (Teerink, 1991; our own reference hair collection). Samples that remained of questionable origins (<1%) were excluded from the analysis.

We prepared and analyzed the fecal samples by means of a standard procedure (Jędrzejewska & Jędrzejewski, 1998). Samples were soaked in water, washed through a sieve (0.5 mm mesh) and dried. All prey remains were separated, and using a microscope, all feather, bone, teeth, hair, fish scales, and seed remains were identified to the lowest possible taxonomic level by using key features (detailed by Lanszki et al., 2020) and our vertebrate, invertebrate and plant reference collections. Besides their identification, we also collected prey frequency data from the individual fecal samples. Small mammals have paired bone structures (e.g., lower jaws, femurs) that allow an assessment of the minimum number of individuals in a scat through the pairing of left and right‐sided bones. Based on the processing, we detected minimum one individual per sample for each prey taxon.

We determined the percentage composition of food items in the scat samples based on the relative frequency of occurrence (RFO, proportion of the total number of occurrences of all items in the sample), frequency of occurrence (FO, proportion of scats containing a given food item), and biomass consumed (BC, the biomass of a given food item expressed as a percentage of the total food biomass consumed). To estimate the fresh mass of food ingested (Reynolds & Aebischer, 1991), all dry food remains were weighed separately (measured at 0.01 g accuracy) and the food remains mass data were multiplied by an appropriate conversion factor (i.e., small mammals by 23, muskrat and carnivores by 50, wild boar by 118, deer by 15, birds and bird eggs by 35, reptiles and amphibians by 18, fish by 25, invertebrates [crayfish, insects, and mollusks] by 5 and plants [fruits, seeds, and other plant material] by 14, as summarized from literature data by Jędrzejewska and Jędrzejewski (1998)).

We used the following 12 food categories in the calculations related to the comparative analysis of the fecal sample composition and the trophic niche or diversity for the two age groups: 1—small rodents, 2—insectivores, 3—muskrat, 4—wild ungulates (i.e., carrion), 5—carnivores, 6—birds, 7—bird eggs, 8—reptiles, 9—amphibians, 10—fish, 11—invertebrates and 12—plants (fruits, seeds, and other plant matter).

Prey size was categorized as either large (>0.5 kg) or small animals (≤0.5 kg) (Lindström, 1994; Table S2). Individual fecal samples were clustered according to whether they contained remains only from large or small prey animals, or species of both types of prey sizes. Consumed prey according to their typical habitat (or zonation, characteristic physical stratification where a species is most active) was classified into characteristically terrestrial and aquatic (species associated with water) groups (e.g., Biró et al., 2005; Gittleman, 1985; Table S2). Individual fecal samples were clustered according to whether they contained only remains from terrestrial, aquatic, or both types of prey species.

### 2.4 Statistical analysis

We used primarily nonparametric statistical tests because the data of examined variables did not show mostly normal distribution with the Shapiro–Wilk test. We used a parametric test (one‐way analysis of variance) to explore the differences in meteorological parameters (mean temperature and precipitation amount of spring months) between the years.

#### 2.4.1 Evaluation of the diet of cubs and adult foxes

We applied general log‐linear analysis on FO data to test for dietary differences within age groups, that is, between adult foxes and cubs, and year (3 years). The unit of analysis was fecal samples of adult and cub foxes from each year and the response variable was the presence or absence of the food item. The model was fitted using age group and year as categories (independent variables). Owing to the large number of comparisons (12 food categories), we adjusted the level of significance to .00417 (*p* = .05/12) with a Bonferroni correction (Lanszki et al., 2007).

To test whether the diet composition based on BC data of individual samples differed between the age groups, we used two‐way permutational multivariate analysis of variance (two‐way PERMANOVA, 9999 random permutations; e.g., Lanszki et al., 2019) with age group and year as the two independent factors, and BC data as dependent factors. Similarity percentage (SIMPER) analysis was applied to highlight which food types contributed most to the dissimilarity in diet composition between the two age groups. PERMANOVA and SIMPER results were based on Bray–Curtis dissimilarity matrices. To visualize the difference in food compositions (BC data) between groups and years, we used hierarchical cluster analysis (Ward method).

We used general log‐linear analysis on the FO data to test for prey weight class and separately for prey habitat differences within age groups and years (we adjusted the significance level to .01666, namely *p* = .05/3, after a Bonferroni correction).

#### 2.4.2 Trophic niche breadth and food diversity analyses

We calculated the trophic niche breadth (B) as described by Levins (1968) and standardized (B_A_; rating from 0 to 1; Hurlbert, 1978). We used various indices to express food variability. The trophic diversity was calculated using the Shannon–Wiener index (Krebs, 1989). Prey species richness represents the number of different animal taxa in samples (e.g., Begg et al., 2003). We used Wilcoxon signed‐rank test on RFO and BC data from each year to test whether the standardized trophic niche breadth and Shannon diversity differed between the age groups, and Mann–Whitney *U*‐test on species richness and on total dry weight per scat samples (pooled years, adult vs. cub samples).

#### 2.4.3 Preference calculation

To calculate the small mammal preference (i.e., was there any preferred sort of small mammal prey from among all small mammals consumed) of foxes we used the Jacobs index (Jacobs, 1974). This formula relates relative prey abundance (dominance data from live trapping [%]); calculated from MNA (minimum number alive) values (Krebs, 1989) to RFO of food items in scats as the measure of diet. In the calculation, the minimum number of consumed prey individuals was considered (in all cases, the known number was one individual per sample). The Jacobs index varies from −1.0 (maximum avoidance) to +1.0 (maximum preference). Each mammal prey (carried to and eaten by cubs) was caught by the fox mother, therefore the small mammal consumption values of adults and cubs were combined. The Jacobs index values were calculated for each small mammal taxa for 3 years, and the small mammal abundance values of each year was compared with the Kruskal–Wallis median test among the prey taxa. When the median test detected significant differences, we employed Dunn's post hoc multiple comparisons procedure. To explore whether there is a difference in “selectivity” that would indicate the quality of parental investment, we compared the small mammal preference values derived from fecal samples of adult foxes and cubs with the Mann–Whitney *U*‐test.

#### 2.4.4 Food energy content estimation

Based on fecal samples, to determine the differences between calculated energetic values of consumed food of adult red foxes and juvenile diets (prey brought to cubs), a simple energy content calculation was applied (Lanszki et al., 2006, 2020). Estimation based on percentage of biomass consumed (BC) in each sample, and metabolizable energy values (KJ/100 g wet weight) for food items detailed in the Table S3. Differences in the calculated energy content of the consumed food depending on the age group and year were examined with a two‐way PERMANOVA (Bray–Curtis similarity index, 9999 permutations).

A minimum probability level of *p* < .05 was accepted in all statistical tests, except log‐linear analyses. Statistical analyses were performed in the program PAST v. 3.20 (Hammer et al., 2001) and R v. 4.1.2 (R Core Team, 2021).

## 3 RESULTS

### 3.1 Diet composition

We identified 566 individual food items in the fecal samples. Among these, small rodents (mainly *Microtus* species and water vole *Arvicola amphibius*) or birds (mainly Anatidae) were the primary food type of foxes (Table 1, Figure S1). Consumption of muskrat (*Ondatra zibethicus*), invertebrates (mostly beetles Coleoptera), bird eggs, and wild ungulate carrion (mainly wild boar *Sus scrofa*) were also considerable in the majority of years, age groups, and calculation methods (RFO, BC). In the fecal samples, the occurrence of the other six food types was occasional (summarized 8.6%, RFO) or had a low proportion (summarized 2.8%, BC) of consumed biomass (Table S2).

With log‐linear analysis of the frequency of occurrence (FO) data of 12 main food types, we found that cubs consumed birds more frequently, while adult foxes consumed more frequently ungulates (Table 2). There were significant differences among years in consumption of small rodents (rarest in Y3), muskrats (most frequent in Y1), birds (all years differed), and invertebrates (rarest in Y3). The age group and year‐dependent differences were not significant for the other food types, nor were the interactions.

**TABLE 2:** Results of log‐linear models for the frequencies of occurrence of food types in the scats of red foxes (*Vulpes vulpes*) during the cub‐rearing period in the Kis‐Balaton marshland (Hungary), for the effect of age group and year, and their interaction.

Item	Effect	*Df*	*χ* ^2^	*p*
Small rodents	Age group	1	2.54	.11111
	Year	2	15.55	**.00042**
	Interaction	2	6.06	.04828
Insectivores	Age group	1	0.70	.40189
	Year	2	1.69	.42929
	Interaction	2	5.00	.08216
Muskrat	Age group	1	0.49	.48451
	Year	2	11.87	**.00042**
	Interaction	2	5.19	.07457
Ungulates	Age group	1	11.60	**.00066**
	Year	2	7.69	.02137
	Interaction	2	5.01	.08172
Carnivores	Age group	1	0.01	.90329
	Year	2	0.34	.84252
	Interaction	2	4.89	.08652
Birds	Age group	1	20.02	**.00001**
	Year	2	12.36	**.00207**
	Interaction	2	4.25	.11923
Bird eggs	Age group	1	0.40	.52593
	Year	2	3.36	.18649
	Interaction	2	5.17	.07555
Reptiles	Age group	1	1.58	.20837
	Year	2	1.08	.58264
	Interaction	2	5.19	.07453
Amphibians	Age group	1	0.86	.35467
	Year	2	1.37	.50418
	Interaction	2	5.15	.07607
Fish	Age group	1	4.22	.03988
	Year	2	3.28	.19393
	Interaction	2	5.01	.08171
Invertebrates	Age group	1	4.90	.02688
	Year	2	15.79	**.00037**
	Interaction	2	6.97	.03061
Plants	Age group	1	1.14	.28473
	Year	2	9.51	.00861
	Interaction	2	5.59	.06114

*Note*: Bolded *p‐*values with Bonferroni corrections indicate significance at the *p* < .00417 level.

**FIGURE 2:**
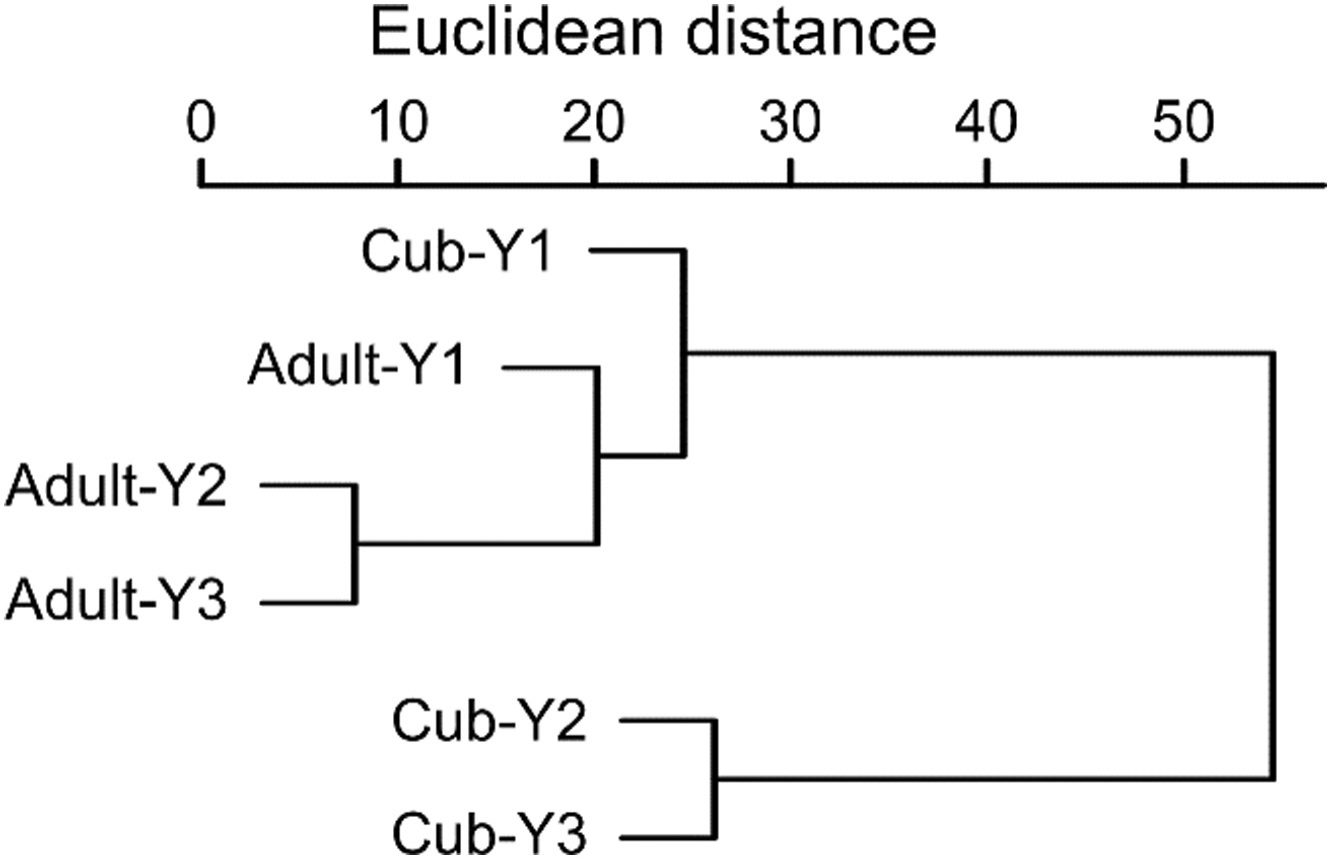
Similarity dendogram of the Euclidean distances among general diet compositions (percentage biomass consumed) of adult and cub red foxes (*Vulpes vulpes*) in the Kis‐Balaton marshland (Hungary). Ward method; 12 main food types; years: Y1–2014, Y2–2017, Y3–2020.

Based on two‐way PERMANOVA analysis of estimated biomass (BC) data, the diet composition of foxes differed significantly between age classes (*F*_1,259_ = 8.14, *p* = .0002) and years (*F*_2,259_ = 6.73, *p* = .0001), and the age class × year interaction was also significant (*F*_2,259_ = 3.32, *p* = .0001). Small rodents and birds (both in higher proportions consumed by cubs), together comprised more than 75% of the difference between diet composition based on age group and year as well (SIMPER, Table S4). The diet composition of cubs in Y2 and Y3, with a high proportion of birds and a low proportion of small mammals differed from the diets of other groups (Figure 2).

The following prey remains were seen around fox dens in Y1: the skull of five muskrats, and in Y3: wings and feathers of three *Porzana* sp. (Rallidae) individuals, one dabbling duck (*Anas* sp.), one undetermined small‐sized waterfowl, three dabbling duck egg shells and scales of one common rudd (*Scardinius erythrophthalmus*).

### 3.2 Food diversity

No significant differences were detected between age groups in the values of standardized trophic niche breadth in the 3 years (Table 1; Wilcoxon signed‐rank test, RFO: *T* = 1.414, *n* = 3, *p* = .157, BC: *T* = 0.000, *n* = 3, *p* = 1.00), the Shannon diversity (Wilcoxon signed‐rank test, RFO: *T* = 0.534, *n* = 3, *p* = .593, BC: *T* = 1.069, *n* = 3, *p* = .285), and prey species richness (Mann–Whitney *U*‐test, *U* = 2.50, *n*_1_ = 3, *n*_2_ = 3, *p* = .501).

### 3.3 Prey size and habitat

**FIGURE 3:**
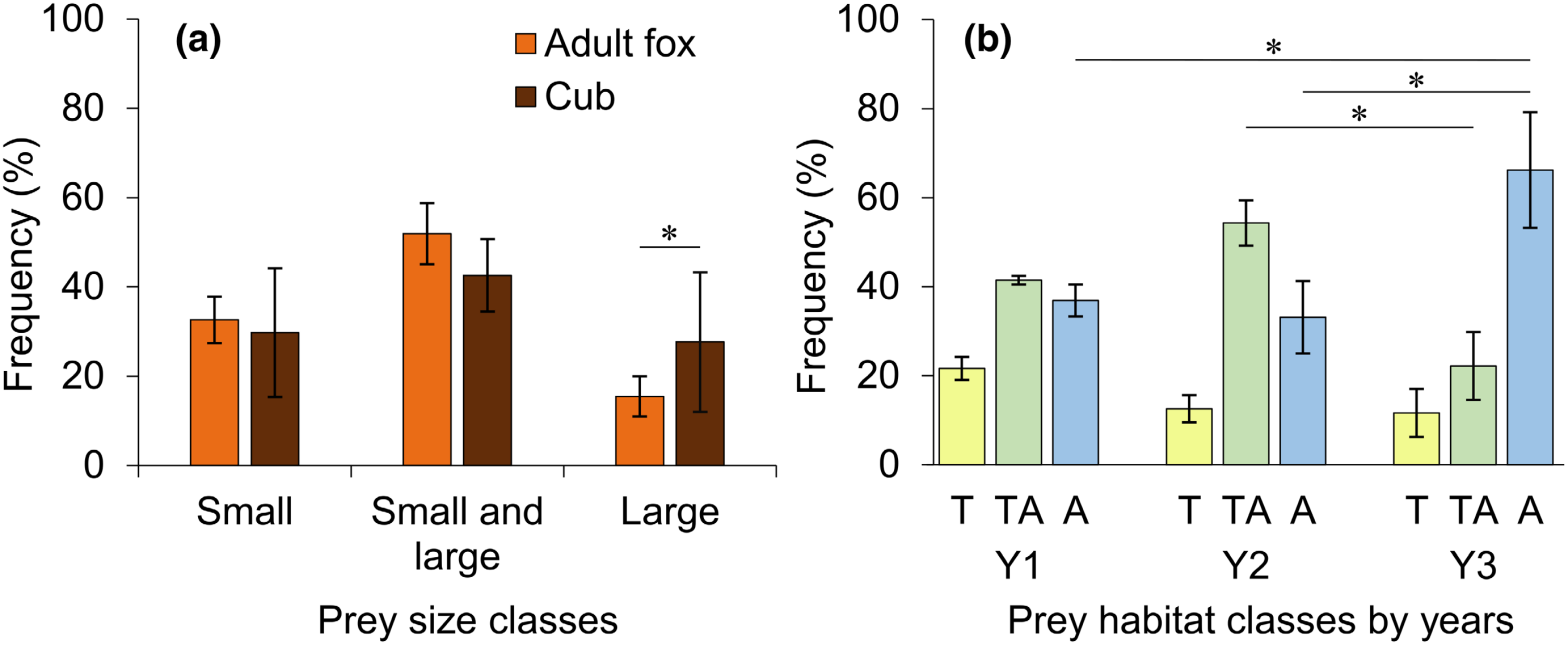
Distribution frequency of prey in fecal samples of red foxes (*Vulpes vulpes*) based on prey size and prey habitat classes in the Kis‐Balaton marshland (Hungary). (a) Prey size classes, small: ≤0.5 kg, large: >0.5 kg. (b) Prey habitat classes: T—terrestrial, A—aquatic, TA—terrestrial and aquatic together. Years: Y1–2014, Y2–2017, Y3–2020. Asterisks indicate significant difference between groups (for significance level see Section 2). Bar values denote mean ± SE.

The age group had a significant association only with the consumption of large‐sized (>0.5 kg) prey. Large preys were consumed more often by cubs (Figure 3a, Table S5). The frequency of consumption of all three prey sizes differed over the years. Foxes consumed small prey significantly more frequently in the Y1, small and large prey together in the Y2, and large prey in the Y3. The age group × year interaction was not significant (Table S5).

**FIGURE 4:**
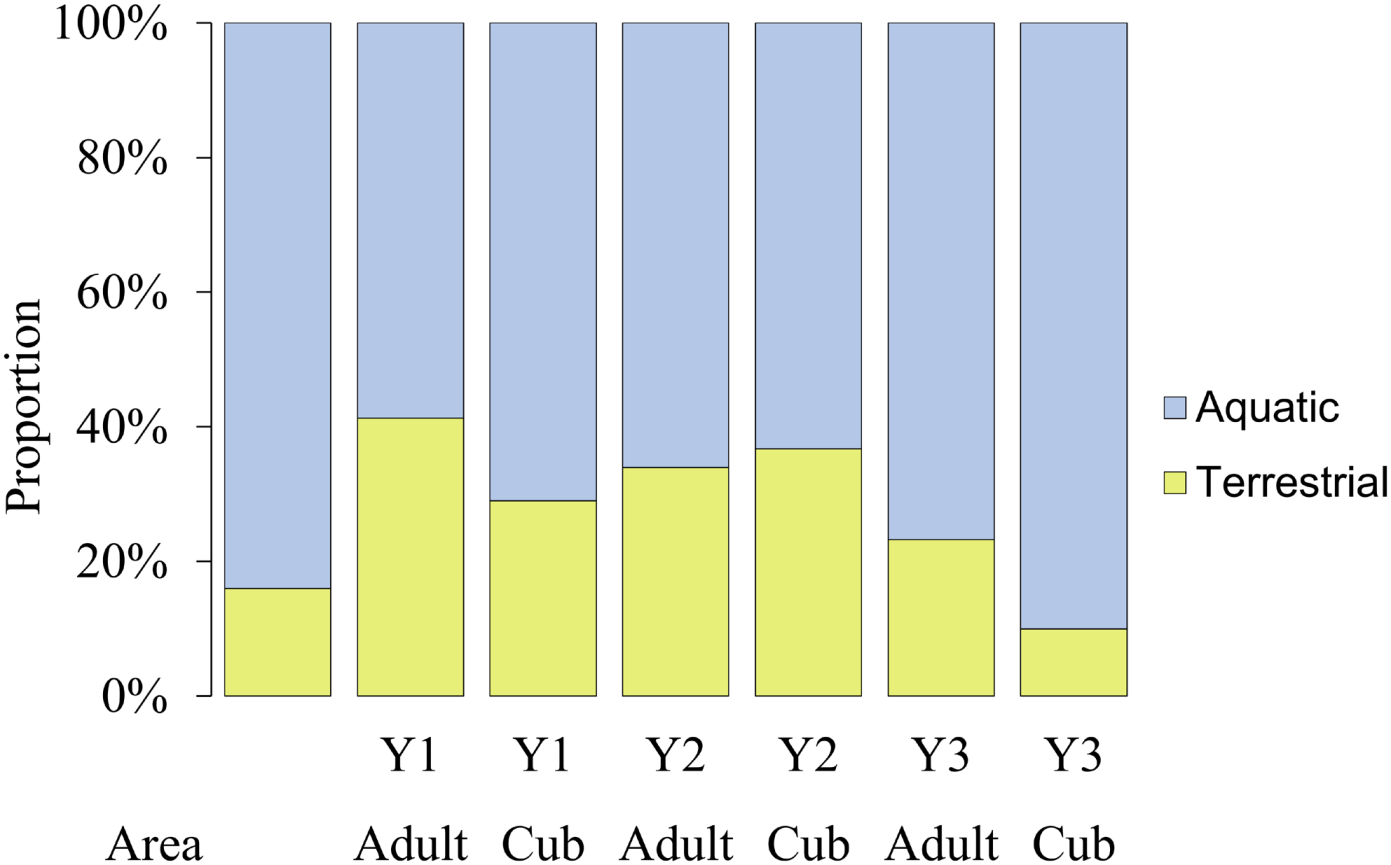
Ratio of terrestrial (yellow) and aquatic (blue) habitats in the Kis‐Balaton marshland and prey species in fecal samples of red foxes (*Vulpes vulpes*) during the cub‐rearing period. The diet composition calculation based on the estimated biomass of consumed food.

No significant age‐dependent differences while significant year‐dependent differences were found in the log‐linear analysis according to the prey habitat (Table S5). The consumption of aquatic prey was significantly more frequent in Y3 than in Y2 (Figure 3b; mean, 66.2% vs. 33.1%) and the combined consumption of aquatic and terrestrial prey was more frequent in Y2 than in Y3 (54.3% vs. 22.2%). The age group × year interaction was not significant (Table S5). Compared with the low proportion of terrestrial habitats (e.g., embankments and islands; 16%), foxes typically consumed relatively higher proportions (BC data) of terrestrial prey (adults: 23.2%–41.3%, cubs: 29.0%–36.7%, with the exception Y3: 10.0%) and aquatic species in a relatively smaller proportion (Figure 4, Table S2).

### 3.4 Food selection/preference

**FIGURE 5:**
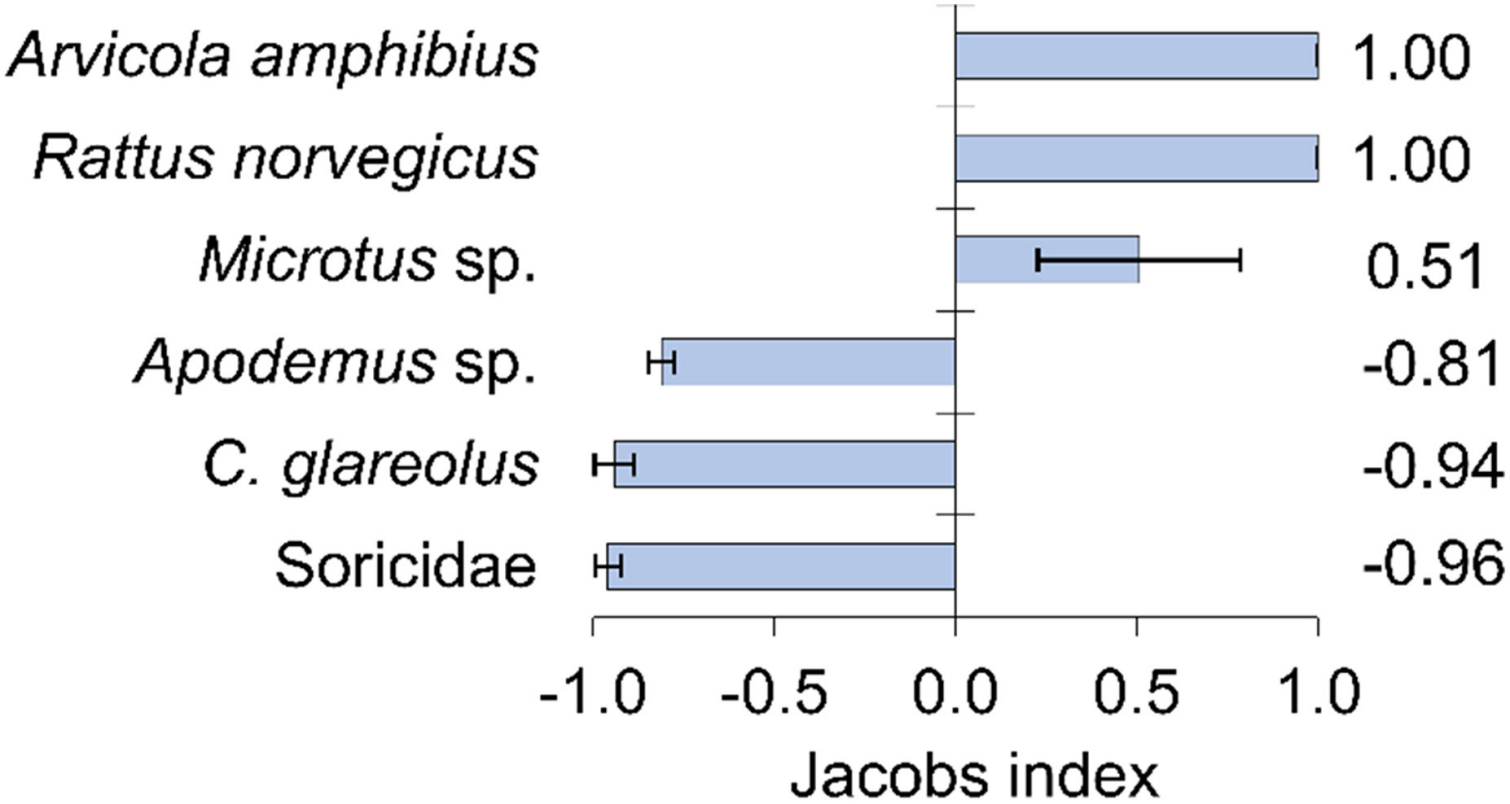
Estimated small mammal selection of red fox (*Vulpes vulpes*) in the Kis‐Balaton marshland (Hungary). Calculated from capture data (small mammal MNA data) and consumption frequency (RFO) values (see Section 2). Error bars represent the standard error of the mean calculated from yearly data.

**FIGURE 6:**
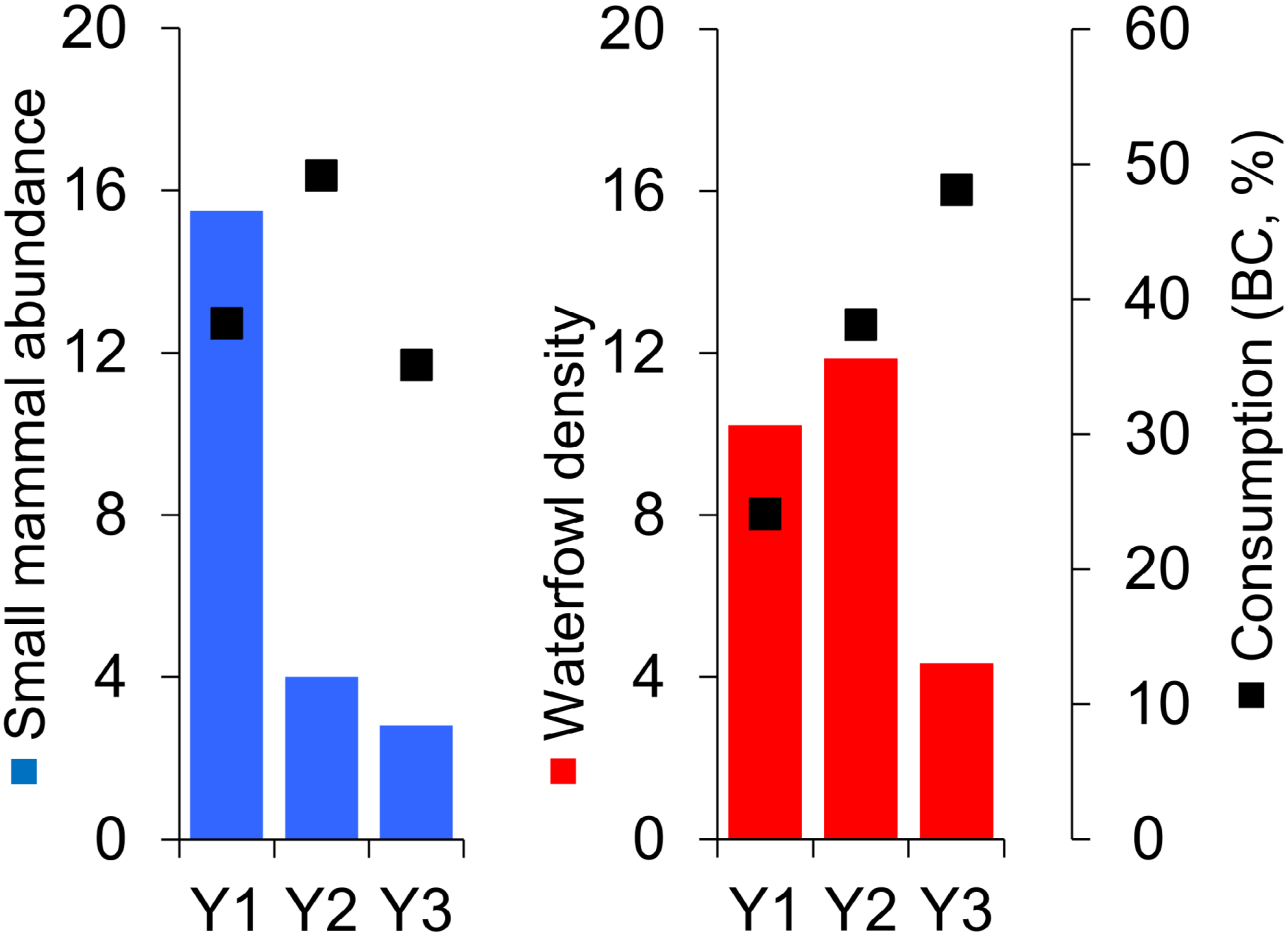
Abundance and consumption (percentage biomass consumed) of main prey taxa of red foxes (*Vulpes vulpes*) in the Kis‐Balaton marshland (Hungary). Small mammal abundance index is based on minimum number alive (MNA) per 100 trap nights obtained by mark–recapture technique in May (source of small mammal MNA data: J. Lanszki). Waterfowl density (individuals/km^2^) data based on direct censuses (direct binocular and spotting scope counting in April and May, source of synchronous bird censuses: Balaton Uplands National Park Directorate's database). Y1–2014, Y2–2017, Y3–2020. Open bars—abundance of small mammals and waterfowl, solid square—percentage consumption by foxes.

Based on small mammal captures (Table S6) and consumption data of combined adult and cub foxes, in addition to *Microtus* species, foxes preferred larger‐sized species, such as water vole (*Arvicola amphibius*) and brown rat (*Rattus norvegicus*), while they avoided the consumption of smaller‐sized rodents and shrews (Figure 5). The difference between the Jacobs preference values of each small mammal taxon was significant (Kruskal–Wallis median test, *χ* ^2^_5_ = 13.33, *p* = .020). There was a significant difference between the preferences of water vole and *Apodemus* mice (*p* = .048), bank vole (*p* = .012) and shrews (*p* = .002), brown rat and shrews (*p* = .032), *Microtus* voles and among the preferences of shrews (*p* = .035). The difference between small mammal preference index values of adult foxes and cubs was not significant (Mann–Whitney *U*‐test, *U* = 127.50, *n*_1_ = 16, *n*_2_ = 16, *p* = 1.00). Foxes consumed a relatively higher percentage of birds and small mammals even when these showed low population densities (Figure 6).

### 3.5 Food energy content

**FIGURE 7:**
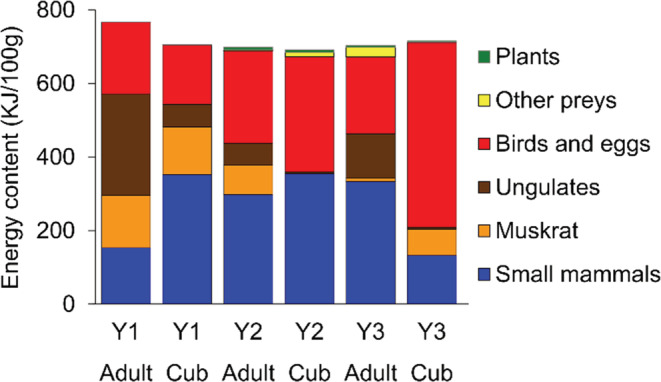
Energetic values of adult and juvenile red fox (*Vulpes vulpes*) diets during the cub‐rearing period in the Kis‐Balaton marshland (Hungary). Estimation based on percentage biomass consumed (Table 1) and details for metabolizable energy calculations can be found in Section 2 and in the Table S3. Years: Y1–2014, Y2–2017, Y3–2020.

Estimated energy values calculated from fox diets based on the scat analysis (Figure 7) did not differ significantly between age groups (two‐way PERMANOVA, *F*_1,259_ = 0.21, *p* = .714) and years (*F*_2,259_ = 1.16, *p* = .290). The interaction between age groups and years was non‐significant (*F*_2,259_ = 1.63, *p* = .285).

## 4 DISCUSSION

Terrestrial mammalian mesopredators, such as the red fox, face a two‐fold challenge when rearing their young, still‐dependent cubs. The constraints of optimal foraging press them to seek the most profitable energy‐rich prey items for self‐sustenance. At the same time, provisioning for the den‐bound cubs with brought back food items requires optimizing for both the logistically most suitable solutions (i.e., the maximum amount of food carried back with the least travel distance) and the best nutrition for the cubs. We predicted that while adult foxes will optimize their own diet according to the actual prey frequencies and abundances of the given year, parental provisioning of the cubs will be maintained according to its own optimal standards, partly independently of the food availabilities. The specific features of the study area (marshland with an almost complete lack of anthropogenic factors and with an abundance of aquatic prey species, especially waterfowl) were expected to add further possibilities for the fox mothers to optimize their cub provisioning behavior.

### 4.1 Differences between the diet of the cubs and adult foxes

In the Kis‐Balaton marshland, the presently discovered differences in dietary patterns among age groups of red foxes as well as among the years, support the theory of distinguished prey selection as part of parental investment. As expected (first prediction), the diet composition of the adult foxes (i.e., prey eaten at the capture site) and dependent cubs (i.e., prey carried to the breeding den for cubs) differed, and the dietary patterns reflected on the temporary and habitat‐based differences in natural sources. Depending on the year (food source), cubs, compared to adult foxes, consumed a higher proportion of the two most important food types, waterfowl, and terrestrial small rodents. Our findings at least partly support the predictions derived from the optimal prey model (Krebs, 1978; Panzacchi et al., 2008; Pyke et al., 1977; Stephens & Krebs, 1986), because even if there were fewer available small mammals or birds, fox mothers still showed a preference toward retrieving these to the cubs.

Prey abundance fluctuations can induce changes in the proportions of alternative prey consumption (Jeschke et al., 2002; Lindström, 1994; Weber, 1996). Importantly, only in the case of self‐sustaining adult foxes we found this behavioral (functional) response, as they switched from a declining (or low) abundance prey to an increasing (or high) abundance prey (Kjellander & Nordström, 2003; Weber, 1996), especially if the alternative prey was also energetically profitable (Carbone et al., 2007; Kruuk, 2006). In our case, adult foxes prominently chose ungulate carcasses in Y1, however, in Y2 and Y3 importance of this food source decreased in the scat samples. Additionally, the proportion of small rodents became larger in the diet of adult foxes, which shows a clear shift of preference, as Y2 and Y3 otherwise were characterized with lower small mammal abundances according to the live trapping surveys. Interestingly, foxes seemingly compensated differently in the case of provisioning their cubs: in Y2 and especially in Y3, the cubs were fed more with avian prey, which can indicate a trade‐off between adult self‐sustenance (biased toward small mammals) and parental provisioning (biased toward waterfowl).

The dominance of birds (mostly waterfowl) and lower consumption of other foods in the cub‐rearing period, differs from the dietary patterns experienced in terrestrial habitat types of Central Europe, where the general dietary dominance of small rodents was described (e.g., Goszczynski, 1986; Jędrzejewska & Jędrzejewski, 1998; Kozená, 1988; Lanszki et al., 2019).

Based on the significant difference between the RFO and BC values, we can assume that the cubs consumed smaller amounts of the large prey several times (Lindström, 1994). The more frequent occurrence of waterfowl and large rodents (muskrat) in the scat samples of fox cubs can be caused by the fact that once caught, these are not necessarily consumed at once (Englund, 1969; Lovari & Parigi, 1995; Soulsbury et al., 2008). This also can explain why these types of prey occur with similar BC values in both age groups of foxes. Cubs may also play with the remains of large‐sized prey; therefore, it can take longer (several feeding occasions) until they are fully consumed. All this, whether it involves already killed or live prey, is part of learning (Biben, 1979; Fairley, 1970; Vincent & Bekoff, 1978). Partly consumed prey remains at the den site cannot be unambiguously assigned to the cubs' diet, however, as adult foxes usually consume their prey at, or near to the catch site, we can assume that any larger prey that occurs at the den was intended to be for the provisioning of the cubs.

The fecal analysis method has limitations, that is, bird egg consumption would be underrepresented (Reynolds & Aebischer, 1991). Still, bird egg consumption was relatively frequent (around 5%, RFO) in both age groups compared with other studies reporting on bird egg consumption in the red fox (2.0%–2.5%, e.g., Jensen & Sequeira, 1978; Kozená, 1988). The egg consumption of cubs was 3.6 times that of adult foxes (BC calculation). Thus, the behavior of foxes in our study follows the predictions of central place foraging theory (Castillo et al., 2011; Orians & Pearson, 1979; Schoener, 1979), namely scavenging from ungulate carcasses on the spot (with low foraging costs), while large‐sized prey (waterfowl, muskrat) and high‐energy content and easy‐to‐transport bird eggs (Careau et al., 2008) are brought to the den for the cubs.

Small mammal remains were equally frequent in the samples from adult foxes and cubs. This indicates the key relevance of this type of prey for the adults' self‐sustenance (local consumption) as well as for the cubs (transported to the den as provision). In line with the CPFT (Orians & Pearson, 1979; Schoener, 1979; Weber, 1996), foxes living in the marshland can be multiple‐prey loaders (in the case of small mammal food; Lloyd, 1980) and single‐prey loaders (in the case of large prey). The body mass of a female mallard is 0.8–1 kg, while that of an *Apodemus* mouse is 20–30 g. According to our study, depending on the current food supply (prey frequency, prey size), the fox can follow both strategies for carrying prey (Weber, 1996). The relatively higher consumption of small‐sized terrestrial prey in terms of habitat type ratio suggests that multiple‐prey loader behaviors may be typical for fox mothers.

The consumption of other food types was of marginal importance or showed a stronger dependency of the year. In the marshland, local consumption of ungulates (scavenging) was characteristic for adult foxes. In contrast, in forested and cultivated areas, fox mothers reportedly carry large animals or their remains (lamb, roe deer fawn, and wild boar) to the cubs (Kolb & Hewson, 1980; Lindström, 1994; Panzacchi et al., 2008; Sidorovich et al., 2015). Differences between the various terrains' suitability for transporting heavier loads by foxes might explain this (Soulsbury et al., 2008).

In our study, domestic animals and human garbage were not present in the samples. Although, according to many publications, Lagomorphs represent a main food source for foxes at various locations (e.g., Castañeda et al., 2022), in marshlands, their relevance may be lower as prey. Invertebrates occurred twice as often in the samples of adult foxes, than in the cubs' samples, but their quantitative proportion was low. Due to their small size and low individual energy content, invertebrates may not be optimal for the fox mothers as transportable food to the cubs (Castillo et al., 2011; Kidawa & Kowalczyk, 2011), despite their high dry matter and protein content. In turn, their consumption by cubs indicates the first independent prey captures (Soulsbury et al., 2008). The low share of plants in the diet may indicate the general and steady abundance of available prey and low availability of profitable plant‐based foods (e.g., fruits) during the cub‐rearing period in this particular area.

### 4.2 Diverse food for cubs

We found highly diverse diets, adult foxes consumed at minimum 61, cubs 53 different food taxa, but their trophic niche was relatively narrow due to the predominance of the two main food types (birds or small rodents) in the diet. Therefore, we did not find differences between age groups in their dietary diversity and could not support our second prediction. The narrow trophic niche of foxes found in this present study could indicate a stable food source in the habitat (Lesser et al., 2020; Ruiz‐Olmo & Jiménez, 2009). This would fit both with the OFT, which states that the generalist consumer would narrow down its food diversity (here trophic niche breadth) in the period of high food abundances (Perry & Pianka, 1997); and with MacArthur's (1955) hypothesis about ecosystem stability, which states that dietary diversity is lower when the habitat is more stable.

### 4.3 Dietary optimization based on prey size, mobility, and habitat

In the marshland, the foxes consumed larger prey more often than small‐sized prey during the cub‐rearing period, but their prey choice based on prey size classes differed each year. This suggests that the female, to provide the cubs with food of optimal prey size, adjusts its preference to the changing supply according to the prey size. In line with our third prediction, we also found that the fox prefers larger prey over smaller ones, even if these were less abundant according to the population survey done in the area. This result fits the predictions of the OFT about the more favorable energy gain/handling time balance in the case of larger prey items (Carbone et al., 2007; Kruuk, 2006).

Red foxes preferred slower‐moving *Microtus* species, bank vole and brown rat, and avoided *Apodemus* mice, which are fast‐moving, very agile species that are difficult to catch, and foul‐smelling shrews (Jędrzejewski & Jędrzejewska, 1992). Similar preferences have been found experimentally (Macdonald, 1977), in terrestrial habitats (Lanszki et al., 2007) and also in marshes (Lanszki, 2005). We did not find age‐related differences in the occurrence of small mammal species in the samples, which suggests that the mother does not choose specifically among these for the cubs.

Although the foxes consumed mostly aquatic prey, the relative occurrence of terrestrial species (by taking into consideration the much smaller ratio of terrestrial habitats) was still almost twice as high. In this particular marshland, the terrestrial habitats are wedged between wetlands (proximity); thus, terrestrial species are easily accessible to the fox. With the decrease in the availability of terrestrial prey (Y3), the foxes switched even more toward the consumption of waterfowl.

### 4.4 Above average energy content of fox diets is independent of the age in the marshland

Analyzing the fecal samples, we did not find significant differences in the total energy content of the food depending on age and year. Our study confirms that foxes compensate by changing/selecting the alternative prey to the optimal energy content of their food and that of their cubs. Despite the food composition variability, the female maintains a balance of supplying herself and her cubs in a natural, diverse environment. Due to the dominance of high‐energy food items (rodents, birds, and bird eggs), the energy content of the fox diets in the studied marshland in May was higher than that of the average energy content of annual diet composition (713.4 KJ/100 g vs. 623.2 KJ/100 g) in Southwest Hungary (Lanszki et al., 2019). It is difficult to determine the optimal value, but the fox can survive and reproduce in less ideal habitats dominated by humans, where food with a much lower specific energy content is typical (e.g., fruit‐dominated diet, seasonal mean: 420.3 KJ/100 g; Lanszki et al., 2020).

### 4.5 Limitations

The limitation of our study is that the capture probabilities of different small mammal taxa could have been different, which could have influenced the calculated preference values. We did not capture brown rats and water voles. Still, at the same time, we detected the presence of these species with camera traps near the waterfront (where the small mammal survey also took place) and their consumption in the current and a previous study in the area (Lanszki et al., 2020). The box traps used in the marsh area can capture black rat (*Rattus rattus*) individuals of different ages, as was experienced in other areas (Lanszki et al., 2016); thus, same traps are probably successful in surveys also on the brown rat.

The detection of the water vole with box traps was occasional in other years. Still, at the same time, it was a particularly common species in the marshland with lower water levels in the Kis‐Balaton (Horváth et al., 2012) in a survey based on the use of box traps. Horváth et al. (2012) used slightly (20 mm) wider plastic box traps (75 mm × 95 mm × 180 mm) than we used and baited traps with cereals as we did. The number of traps used per sampling unit (on five transects *n* = 50 traps per transect) could have been minimally limiting compared with the low numbers of catches (Table S1). Due to the expected limitations of the trappability of large‐sized water vole individuals, the preference index value can be lower than +1.

## 5 CONCLUSION

We found parallel optimizing foraging strategies: adult foxes optimized their diet according to the temporal (yearly) fluctuation of the abundance of main prey types; meanwhile, fox mothers kept provisioning their cubs with optimal size and energy content prey types even if they became less abundant. According to this, for self‐sustenance, adult foxes consumed small mammals and ungulate carrion in times of relative abundance; but kept on carrying home large numbers of small mammals at a time even in those years when these were less abundant. Additionally, adult fox scats show a preference for prey items that are consumed at the site of finding it (large carrion and small individual prey, such as invertebrates), but cubs were provisioned with prey that has a more optimal energy content/carrying cost balance (such as waterfowl and muskrat, plus multiple small mammals in one load).

The specific features of marshland habitat are characterized by the lack of domestic animals, human‐originated garbage and crops and Lagomorphs that otherwise would be typical as food for foxes. In turn, the abundance of waterfowl and muskrat offers an optimal choice for provisioning the fox cubs, providing both an energy‐rich choice for fox mothers and a potential source of learning how to handle prey for the cubs.

## AUTHOR CONTRIBUTIONS

**József Lanszki:** Conceptualization (equal); data curation (lead); formal analysis (lead); methodology (equal); writing – original draft (equal); writing – review and editing (equal). **Zsolt Bende:** Conceptualization (supporting); formal analysis (supporting); methodology (equal); writing – review and editing (supporting). **Nikolett Nagyapáti:** Conceptualization (supporting); data curation (supporting); writing – review and editing (supporting). **Zsófia Lanszki:** Data curation (supporting); visualization (lead); writing – review and editing (supporting). **Péter Pongrácz:** Conceptualization (equal); formal analysis (supporting); supervision (lead); writing – original draft (equal); writing – review and editing (equal).

## CONFLICT OF INTEREST STATEMENT

The authors declare no competing interests.

## Supporting information


Appendix S1.

## Data Availability

Raw dataset is available at: https://doi.org/10.5061/dryad.cfxpnvx9r.
