# Supplementary material for: Optimal prey for red fox cubs—An example of dual optimizing foraging strategy in foxes from a dynamic wetland habitat
Source: Ecol Evol. 2023 Apr 20;13(4):e10033. doi: 10.1002/ece3.10033 (PMC10119026; doi:10.1002/ece3.10033)
Supplement: Supplementary file 1 — Appendix S1. [file ECE3-13-e10033-s001.doc]

**Appendices**

Journal: *Ecology and Evolution*

Title:

**Optimal prey for red fox cubs – an example of dual optimizing foraging strategy in foxes from a dynamic wetland habitat**

Authors: József Lanszki^1^, Zsolt Bende^2^, Nikolett Nagyapáti^3^, Zsófia Lanszki^4^, Péter Pongrácz^5*^

Affiliations:

^1^Fish and Conservation Ecology Research Group, Balaton Limnological Research Institute, Tihany, Hungary

^2^Balaton Uplands National Park Directorate, Csopak, Hungary

^3^Duna-Ipoly National Park Directorate, Budapest, Hungary

^4^Institute of Biology, University of Pécs, Pécs, Hungary

^5^Department of Ethology, ELTE Eötvös Loránd University, Budapest, Hungary

^*^Corresponding author. E-mail: [peter.pongracz@ttk.elte.hu](mailto:peter.pongracz@ttk.elte.hu)

**Table A1** Statistics of differences in small mammal abundance between years by Kruskal-Wallis median test. MNA – abundance of small mammals (minimum number alive/100 trap nights), *p*-values from Dunn post-hoc test in boldface are significant. Traps were placed in dense vegetation along the foot of the embankment on dry soil. Glass-doored wooden box-type live-traps (size 180×70×70 mm; Lanszki et al. 2007) were distributed every 10 metres on five lines (50 traps/line, 500-meter-long lines), baited with walnuts and maize. We checked the traps twice a day at 7:00 and 19:00 (8 consecutive checks in each session). To identify captured animals, we used non-individualized fur clipping on the head.

| Year | MNA |  | Y1 | Y2 | Y3 |
| --- | --- | --- | --- | --- | --- |
|  | small |  | 2014 | 2017 | 2020 |
|  | mammals |  | *p*-value | |  |
| 2010 | 10.9 |  | 0.5076 | **0.0493** | **0.0125** |
| 2011 | 7.6 |  | 0.1851 | 0.1924 | 0.0664 |
| 2013 | 8.3 |  | 0.2774 | 0.1230 | **0.0380** |
| 2014 | 15.5 |  |  | **0.0086** | **0.0016** |
| 2015 | 8.6 |  | 0.4029 | 0.0731 | **0.0201** |
| 2016 | 4.4 |  | **0.0141** | 0.8620 | 0.4802 |
| 2017 | 4.0 |  | **0.0086** |  | 0.5945 |
| 2018 | 1.5 |  | **0.0002** | 0.2970 | 0.6097 |
| 2019 | 6.2 |  | 0.0766 | 0.3908 | 0.1644 |
| 2020 | 2.8 |  | **0.0016** | 0.5945 |  |

**Table A2** Diet composition of adult and cub red foxes (*Vulpes vulpes*) based on faecal samples in the Kis-Balaton marshland (Hungary). Weight classes: 1 – small (≤ 0.5 kg), 2 – large (> 0.5 kg), habitat type classes: t – terrestrial, a – aquatic. Faecal samples were collected during the cub rearing period in Y1 (first year – May 2014), Y2 (second year – May 2017) and Y3 (third year – May 2020). *N* – number of food items per taxon. BC – estimated biomass of consumed food, + – biomass < 0.05%, juv. – juvenile, indet. – indeterminable.

| Food items | Weight | Habitat |  | Y1 |  |  |  |  |  | Y2 |  |  |  |  |  | Y3 |  |  |  |  |
| --- | --- | --- | --- | --- | --- | --- | --- | --- | --- | --- | --- | --- | --- | --- | --- | --- | --- | --- | --- | --- |
|  | class | type |  | Adult | |  | Cub |  |  | Adult | |  | Cub |  |  | Adult | |  | Cub | |
|  |  | class |  | N | BC |  | N | BC |  | N | BC |  | N | BC |  | N | BC |  | N | BC |
| Common vole (*Microtus arvalis*) | 1 | t |  | 1 | 0.3 |  |  |  |  | 4 | 6.6 |  | 1 | 0.6 |  |  |  |  |  |  |
| Field vole (*Microtus agrestis*) | 1 | t |  |  |  |  |  |  |  | 2 | 3.1 |  |  |  |  | 1 | 1.9 |  |  |  |
| *Microtus* sp. | 1 | t |  | 9 | 8.4 |  | 13 | 13.0 |  | 12 | 13.2 |  | 27 | 27.4 |  | 6 | 2.2 |  | 3 | 0.9 |
| Bank vole (*Clethrionomys glareolus*) | 1 | t |  |  |  |  |  |  |  | 1 | 1.1 |  |  |  |  |  |  |  |  |  |
| European water vole (*Arvicola amphibius*) | 1 | a |  | 11 | 13.2 |  | 19 | 31.5 |  | 6 | 19.5 |  | 12 | 20.5 |  | 21 | 40.9 |  | 11 | 13.3 |
| Field mice (*Apodemus* sp.) | 1 | t |  | 1 | 0.2 |  | 7 | 8.6 |  | 3 | 1.2 |  | 4 | 5.6 |  | 1 | + |  | 1 | 4.0 |
| Brown rat (*Rattus norvegicus*) | 1 | t |  |  |  |  |  |  |  |  |  |  |  |  |  | 3 | 5.2 |  | 2 | 1.7 |
| Shrews (Soricidae) | 1 | a |  |  |  |  |  |  |  |  |  |  | 1 | + |  |  |  |  |  |  |
| European mole (*Talpa europaea*) | 1 | t |  | 2 | 1.2 |  |  |  |  |  |  |  |  |  |  |  |  |  |  |  |

Table A2 – continuation

| Food items | Weight | Habitat |  | Y1 |  |  |  |  |  | Y2 |  |  |  |  |  | Y3 |  |  |  |  |
| --- | --- | --- | --- | --- | --- | --- | --- | --- | --- | --- | --- | --- | --- | --- | --- | --- | --- | --- | --- | --- |
|  | class | type |  | Adult | |  | Cub |  |  | Adult | |  | Cub |  |  | Adult | |  | Cub | |
|  |  | class |  | N | BC |  | N | BC |  | N | BC |  | N | BC |  | N | BC |  | N | BC |
| Muskrat (*Ondatra zibethicus*) | 2 | a |  | 4 | 19.9 |  | 7 | 18.0 |  | 1 | 11.1 |  |  |  |  | 1 | 1.3 |  | 4 | 10.0 |
| Stoat (*Mustela erminea*) | 1 | t |  |  |  |  |  |  |  |  |  |  | 1 | 1.7 |  |  |  |  |  |  |
| Wild boar (*Sus scrofa*) | 2 | t |  | 1 | 0.4 |  | 2 | 0.7 |  | 2 | 0.3 |  |  |  |  | 1 | 0.1 |  |  |  |
| Wild boar (*Sus scrofa*), juv. | 2 | t |  | 9 | 30.0 |  | 3 | 6.1 |  | 1 | 6.2 |  |  |  |  | 2 | 11.8 |  |  |  |
| Roe deer (*Capreolus capreolus*) | 2 | t |  |  |  |  |  |  |  | 2 | 0.2 |  | 1 | 0.6 |  | 2 | 0.7 |  | 1 | 0.6 |
| Red deer (*Cervus elaphus*) | 2 | t |  |  |  |  |  |  |  |  |  |  | 1 | + |  | 2 | 0.9 |  |  |  |
| Small passerines (Passeriformes), indet. | 1 | t |  | 2 | 0.9 |  | 1 | 0.5 |  | 1 | 1.0 |  | 1 | + |  |  |  |  | 3 | 1.8 |
| Common pheasant (Phasianus colchicus) | 2 | t |  |  |  |  |  |  |  |  |  |  |  |  |  |  |  |  | 1 | 0.8 |
| Rails (Rallidae), indet. | 1 | a |  | 2 | 0.3 |  |  |  |  |  |  |  | 1 | 0.2 |  |  |  |  |  |  |
| Great cormorant (*Phalacrocorax carbo*) | 2 | a |  |  |  |  | 1 | 0.1 |  |  |  |  |  |  |  |  |  |  |  |  |
| Loons (*Gavia* sp.) | 2 | a |  | 1 | 0.3 |  |  |  |  |  |  |  |  |  |  |  |  |  |  |  |
| Mute swan (*Cygnus olor*) | 2 | a |  | 6 | 4.4 |  | 4 | 12.7 |  | 1 | 4.1 |  | 19 | 6.3 |  |  |  |  |  |  |
| Great egret (*Ardea alba*) | 2 | a |  |  |  |  |  |  |  |  |  |  | 1 | 1.1 |  |  |  |  |  |  |

Table A2 – continuation

| Food items | Weight | Habitat |  | Y1 |  |  |  |  |  | Y2 |  |  |  |  |  | Y3 |  |  | |  | |  | |
| --- | --- | --- | --- | --- | --- | --- | --- | --- | --- | --- | --- | --- | --- | --- | --- | --- | --- | --- | --- | --- | --- | --- | --- |
|  | class | type |  | Adult | |  | Cub |  |  | Adult | |  | Cub |  |  | Adult | |  | | Cub | | | |
|  |  | class |  | N | BC |  | N | BC |  | N | BC |  | N | BC |  | N | BC |  | | N | | BC | |
| Egret (*Ardea alba/Egretta garzetta*) | 2 | a |  |  |  |  |  |  |  |  |  |  | 1 | 1.0 |  |  |  |  | |  | |  | |
| Grey heron (*Ardea cinerea*) | 2 | a |  |  |  |  |  |  |  |  |  |  |  |  |  |  |  |  | 1 | | 3.1 | |  |
| Herons (Ardeidae), indet. | 2 | a |  |  |  |  |  |  |  |  |  |  | 3 | 1.0 |  | 1 | 0.7 |  | | 10 | | 8.7 | |
| Mallard (*Anas platyrhynchos*), female | 2 | a |  |  |  |  |  |  |  |  |  |  | 3 | 3.2 |  |  |  |  | |  | |  | |
| Mallard (*Anas platyrhynchos*), male | 2 | a |  |  |  |  |  |  |  |  |  |  | 1 | 2.4 |  |  |  |  | |  | |  | |
| Mallard (*Anas platyrhynchos*), adult | 2 | a |  |  |  |  |  |  |  |  |  |  | 3 | 2.0 |  | 9 | 16.4 |  | | 6 | | 11.6 | |
| Dabbling ducks (*Anas* sp.), male | 2 | a |  |  |  |  |  |  |  | 1 | 10.3 |  |  |  |  |  |  |  | |  | |  | |
| Dabbling ducks (*Anas* sp.), adult | 2 | a |  | 8 | 19.3 |  | 5 | 6.7 |  | 8 | 9.1 |  | 29 | 18.7 |  |  |  |  | | 18 | | 27.3 | |
| Dabbling ducks (*Anas* sp.), juv. | 2 | a |  |  |  |  |  |  |  |  |  |  |  |  |  | 2 | 6.8 |  | | 4 | | 3.9 | |
| Mute swan (*Cygnus olor*) | 2 | a |  |  |  |  |  |  |  |  |  |  |  |  |  | 5 | 3.7 |  | 4 | | 3.4 | |  |
| Eurasian coot (*Fulica atra*) | 2 | a |  |  |  |  | 4 | 1.3 |  | 3 | 4.0 |  | 1 | + |  |  |  |  | |  | |  | |
| Great crested grebe (*Podiceps cristatus*) | 2 | a |  |  |  |  |  |  |  |  |  |  |  |  |  |  |  |  | | 1 | | 4.0 | |
| Grebes (Podicipedidae) | 2 | a |  |  |  |  |  |  |  | 1 | 2.3 |  |  |  |  |  |  |  | |  | |  | |

Table A2 – continuation

| Food items | Weight | Habitat |  | Y1 |  |  |  |  |  | Y2 |  |  |  |  |  | Y3 |  |  |  |  |
| --- | --- | --- | --- | --- | --- | --- | --- | --- | --- | --- | --- | --- | --- | --- | --- | --- | --- | --- | --- | --- |
|  | class | type |  | Adult | |  | Cub |  |  | Adult | |  | Cub |  |  | Adult | |  | Cub | |
|  |  | class |  | N | BC |  | N | BC |  | N | BC |  | N | BC |  | N | BC |  | N | BC |
| Waterfowls (Anatidae) | 2 | a |  |  |  |  |  |  |  |  |  |  |  |  |  |  |  |  | 3 | 0.2 |
| Medium sized birds (Aves) | 2 | a |  | 1 | + |  |  |  |  | 5 | 3.2 |  | 4 | 1.8 |  | 4 | 0.1 |  |  |  |
| Rails (Rallidae), egg | 1 | a |  | 1 | 0.1 |  |  |  |  |  |  |  | 1 | 0.3 |  |  |  |  |  |  |
| Dabbling ducks (Anas sp.), egg | 1 | a |  | 1 | 0.3 |  |  |  |  |  |  |  | 6 | 2.8 |  |  |  |  |  |  |
| Birds (Aves), nestling | 1 | a |  |  |  |  | 2 | 0.2 |  |  |  |  |  |  |  |  |  |  |  |  |
| Birds (Aves), indet. | 1 | a |  |  |  |  | 6 | 0.4 |  |  |  |  | 1 | 0.2 |  |  |  |  |  |  |
| Bird egg | 1 | a |  | 7 | 0.8 |  |  |  |  | 3 | 0.1 |  | 5 | 0.3 |  | 2 | 0.6 |  | 5 | 3.1 |
| Colubrids (Colubridae), indet. | 1 | a |  | 1 | + |  |  |  |  |  |  |  |  |  |  |  |  |  |  |  |
| Lizards (Sauria), indet. | 1 | t |  | 1 | + |  |  |  |  | 1 | + |  |  |  |  |  |  |  |  |  |
| European pond turtle (E*mys orbicularis*), egg | 1 | a |  |  |  |  |  |  |  |  |  |  |  |  |  |  |  |  | 1 | 0.1 |
| Reptiles (Reptilia) | 1 | a |  |  |  |  |  |  |  | 1 | + |  | 1 | + |  |  |  |  |  |  |
| True frogs (*Rana* sp.) | 1 | a |  |  |  |  |  |  |  | 1 | + |  | 1 | + |  |  |  |  |  |  |
| Anurans (Anura), indet. | 1 | a |  | 1 | + |  |  |  |  |  |  |  |  |  |  |  |  |  |  |  |

Table A2 – continuation

| Food items | Weight | Habitat |  | Y1 |  |  |  |  |  | Y2 |  |  |  |  |  | Y3 |  |  |  |  |
| --- | --- | --- | --- | --- | --- | --- | --- | --- | --- | --- | --- | --- | --- | --- | --- | --- | --- | --- | --- | --- |
|  | class | type |  | Adult | |  | Cub |  |  | Adult | |  | Cub |  |  | Adult | |  | Cub | |
|  |  | class |  | N | BC |  | N | BC |  | N | BC |  | N | BC |  | N | BC |  | N | BC |
| Gibel carp (*Carassius gibelio*) | 1 | a |  |  |  |  |  |  |  |  |  |  |  |  |  | 1 | 4.1 |  |  |  |
| Common carp (*Cyprinus carpio*) | 2 | a |  |  |  |  |  |  |  |  |  |  |  |  |  | 1 | 0.6 |  |  |  |
| Cyprinids (Cyprinidae), < 0.1 kg | 1 | a |  |  |  |  |  |  |  | 2 | 0.4 |  | 1 | + |  | 2 | 0.4 |  | 1 | + |
| Fish (Pisces), indet., < 0.1 kg | 1 | a |  |  |  |  |  |  |  | 1 | + |  |  |  |  |  |  |  |  |  |
| European mole cricket (*Gryllotalpa gryllotalpa*) | 1 | t |  | 2 | + |  |  |  |  |  |  |  |  |  |  |  |  |  |  |  |
| Carabid beetles (Carabidae) | 1 | t |  | 1 | + |  |  |  |  | 1 | + |  |  |  |  |  |  |  |  |  |
| Water beetles (Dytiscidae, Hydrophilidae) | 1 | a |  | 1 | + |  |  |  |  | 5 | + |  | 6 | 0.1 |  | 1 | + |  |  |  |
| Rose chafer (Cetonia) | 1 | t |  |  |  |  | 1 | + |  |  |  |  |  |  |  |  |  |  |  |  |
| Longhorn beetles (Cerambycidae) | 1 | t |  | 1 | + |  |  |  |  |  |  |  |  |  |  |  |  |  |  |  |
| Ground beetles (*Pterostichus* spp.) | 1 | t |  |  |  |  |  |  |  |  |  |  |  |  |  | 1 | + |  |  |  |
| Scarab beetles (Scarabaeidae) | 1 | t |  |  |  |  |  |  |  |  |  |  | 5 | + |  | 1 | + |  |  |  |
| Cockchafers (Melolonthinae) | 1 | t |  | 1 | + |  |  |  |  |  |  |  | 1 | + |  | 1 | + |  |  |  |

Table A2 – continuation

| Food items | Weight | Habitat |  | Y1 |  |  |  |  |  | Y2 |  |  |  |  |  | Y3 |  |  |  |  |
| --- | --- | --- | --- | --- | --- | --- | --- | --- | --- | --- | --- | --- | --- | --- | --- | --- | --- | --- | --- | --- |
|  | class | type |  | Adult | |  | Cub |  |  | Adult | |  | Cub |  |  | Adult | |  | Cub | |
|  |  | class |  | N | BC |  | N | BC |  | N | BC |  | N | BC |  | N | BC |  | N | BC |
| Beetles (Coleoptera), indet. | 1 | t |  | 1 | + |  | 1 | + |  |  |  |  | 7 | + |  | 2 | + |  |  |  |
| Wasps (Vespidae) | 1 | t |  |  |  |  | 1 | + |  |  |  |  | 1 | + |  |  |  |  |  |  |
| Hymenopterans (Hymenoptera) | 1 | t |  |  |  |  |  |  |  | 1 | + |  |  |  |  |  |  |  |  |  |
| Net-winged insects (Neuroptera) | 1 | t |  |  |  |  |  |  |  |  |  |  | 1 | + |  |  |  |  |  |  |
| Dragonfly (Odonata) | 1 | t |  | 1 | + |  |  |  |  |  |  |  |  |  |  |  |  |  |  |  |
| Heteropterans (Heteroptera) | 1 | t |  |  |  |  | 1 | + |  |  |  |  |  |  |  |  |  |  |  |  |
| Insects (Insecta), indet. | 1 | t |  | 2 | + |  |  |  |  | 1 | + |  |  |  |  |  |  |  |  |  |
| Insects (Insecta), larvae | 1 | t |  | 1 | + |  |  |  |  | 1 | + |  |  |  |  | 1 | 0.1 |  |  |  |
| Marbled crayfish (*Procambarus fallax f. virginalis*) | 1 | a |  |  |  |  |  |  |  | 1 | + |  |  |  |  |  |  |  |  |  |
| Blackthorn (*Prunus spinosa*) |  |  |  |  |  |  | 1 | 0.2 |  |  |  |  |  |  |  |  |  |  |  |  |
| Plum (*Prunus domestica*) |  |  |  |  |  |  |  |  |  |  |  |  |  |  |  | 2 | 1.2 |  |  |  |
| Cherry (*Prunus* sp.) |  |  |  |  |  |  |  |  |  | 2 | 2.0 |  |  |  |  |  |  |  |  |  |

Table A2 – concluded

| Food items |  |  |  | Y1 |  |  |  |  |  | Y2 |  |  |  |  |  | Y3 |  |  |  |  |
| --- | --- | --- | --- | --- | --- | --- | --- | --- | --- | --- | --- | --- | --- | --- | --- | --- | --- | --- | --- | --- |
|  |  |  |  | Adult | |  | Cub |  |  | Adult | |  | Cub |  |  | Adult | |  | Cub | |
|  |  |  |  | N | BC |  | N | BC |  | N | BC |  | N | BC |  | N | BC |  | N | BC |
| Maize (*Zea mays*) |  |  |  |  |  |  |  |  |  | 2 | 0.7 |  | 8 | 1.9 |  |  |  |  |  |  |
| Sunflower (*Helianthus annuus*) |  |  |  |  |  |  |  |  |  |  |  |  | 1 | + |  |  |  |  |  |  |
| Other seeds |  |  |  |  |  |  |  |  |  | 1 | + |  | 2 | + |  | 1 | 0.4 |  |  |  |
| Grass |  |  |  | 2 | + |  |  |  |  | 1 | + |  | 1 | + |  | 2 | + |  | 2 | 1.3 |
| Number of samples analysed |  |  |  | 33 |  |  | 42 |  |  | 32 |  |  | 63 |  |  | 47 |  |  | 48 |  |
| Number of items |  |  |  | 83 |  |  | 79 |  |  | 79 |  |  | 164 |  |  | 79 |  |  | 82 |  |
| Samples with small prey only |  |  |  | 10 |  |  | 24 |  |  | 8 |  |  | 15 |  |  | 20 |  |  | 4 |  |
| Samples with large prey only |  |  |  | 8 |  |  | 3 |  |  | 3 |  |  | 11 |  |  | 6 |  |  | 28 |  |
| Samples with small and large prey |  |  |  | 15 |  |  | 15 |  |  | 21 |  |  | 37 |  |  | 21 |  |  | 16 |  |
| Samples with terrestrial prey only |  |  |  | 8 |  |  | 8 |  |  | 5 |  |  | 6 |  |  | 8 |  |  | 3 |  |
| Samples with aquatic prey only |  |  |  | 11 |  |  | 17 |  |  | 8 |  |  | 26 |  |  | 25 |  |  | 38 |  |
| Samples with terrestrial and aquatic prey |  |  |  | 14 |  |  | 17 |  |  | 19 |  |  | 31 |  |  | 14 |  |  | 7 |  |

**Table A3** Energy content (KJ/100 g wet weight) of different foods considered in the fox dietary study based on literature data

| Taxon | KJ/100g | Source |
| --- | --- | --- |
| Small rodents (common vole *Microtus arvalis*; summer period) | 663.5 | Górecki (1965) |
| Insectivores (common shrew *Sorex araneus*; summer period) | 572.8 | Górecki (1965) |
| Muskrat (*Ondatra zibethicus*; spring period) | 717.4 | Virgl and Messier (1992) |
| Ungulates (roe deer *Capreolus capreolus* carcass) | 905.5 | Weiner (1973) |
| Passerines (European tree sparrow *Passer montanus*) | 825.0 | Myrcha and Pinowski (1970) |
| Other birds/waterfowl (mallard duck *Anas platyrhynchos*) | 736.6 | Lanszki et al. (2006) |
| Eggs (duck egg) | 795.5 | Ricklefs (1977) |
| Reptiles (European pond turtle *Emys orbicularis*; mean value of consumable body parts) | 402.2 | Lanszki et al. (2006) |
| Amphibians (edible frog *Rana* kl. *esculenta*) | 348.9 | Lanszki et al. (2006) |
| Fish (gibel carp *Carassius gibelio*) | 491.9 | Lanszki et al. (2006) |
| Insects (beetles, *Polycleis* spp., *Sternocera* spp., imago) | 806.4 | Bukkens (1997) |
| Plants (a mixture of corn *Zea mays*, blackberry *Prunus spinosa* and plum *Prunus domestica*) | 285.7 | U.S. Department of Agriculture (2019) |

References to Table A3

Bukkens SG (1997) The nutritional value of edible insects. Ecol Food Nutr 36:287–319. https://doi.org/10.1080/03670244.1997.9991521

Górecki A (1965) Energy values of body in small mammals. Acta Theriol 10:333–352.

U.S. Department of Agriculture (2019) Agricultural Research Service. Food Data Central, <https://ndb.nal.usda.gov/ndb>

Lanszki J, Molnár M, Molnár T (2006) Factors affecting the predation of otter (*Lutra lutra*) on European pond turtle (*Emys orbicularis*). J Zool 270:219–226. https://doi.org/10.1111/j.1469-7998.2006.00132.x

Myrcha A, Pinowski J (1970) Weights, body composition, and caloric value of postjuvenal molting European Tree Sparrows (*Passer montanus*). Condor 72:175–181. <https://doi.org/10.2307/1366628>

Ricklefs RE (1977) Composition of eggs of several bird species. Auk 94:350–356. https://doi.org/10.1093/auk/94.2.350

Virgl JA, Messier F (1992) Seasonal variation in body composition and morphology of adult muskrats in central Saskatchewan, Canada. J Zool 228:461–477. https://doi.org/10.1111/j.1469-7998.1992.tb04449.x

Weiner J (1973) Dressing percentage, gross body composition and caloric value of the roe-deer. Acta Theriol 18:209–222.

**Table A4** Food categories (BC) with the highest impact on differences in the diet of red foxes living in the Kis-Balaton marshland, depending on age group and year (SIMPER analysis). BC – estimated biomass of consumed food.

| Food type | Age | Year |  |  |
| --- | --- | --- | --- | --- |
|  | group | 1–2 | 1–3 | 2–3 |
|  | Contribution % | | | |
| Small rodents | 38.5 | 39.7 | 38.6 | 39.6 |
| Birds | 36.7 | 35.6 | 35.5 | 39.2 |
| **Small rodents and birds together** | **75.3** | **75.4** | **74.1** | **78.8** |
| Ungulates | 9.8 | 8.4 | 10.7 | 7.0 |
| Muskrat | 6.3 | 8.3 | 8.9 | 3.6 |
| Plants | 3.0 | 3.0 | 1.5 | 4.4 |
| Fish | 2.0 | 0.3 | 2.0 | 2.5 |
| Bird eggs | 1.9 | 2.2 | 1.2 | 2.6 |
| Insectivores | 0.9 | 1.4 | 1.3 | 0.0 |
| Carnivores | 0.4 | 0.6 | 0.0 | 0.6 |
| Invertebrates | 0.2 | 0.2 | 0.2 | 0.3 |
| Reptiles | 0.1 | 0.1 | 0.1 | 0.2 |
| Amphibians | 0.1 | 0.1 | 0.03 | 0.1 |
| Overall average dissimilarity (%) | 62.8 | 59.8 | 66.0 | 66.4 |

**Table A5** Results of log-linear models for the frequencies of occurrence of prey size and prey habitat classes in the scats of red foxes (*Vulpes vulpes*) during cub rearing period in the Kis-Balaton marshland (Hungary), for the effect of age group and year, and their interaction. Bolded *p-*values with Bonferroni corrections indicate signiﬁcance at the *p* < 0.01666 level.

| Prey characteristic | Effect | *df* | *χ*^2^ | *p* |
| --- | --- | --- | --- | --- |
| Size class |  |  |  |  |
| Small | Age group | 1 | 0.92 | 0.33862 |
|  | Year | 2 | 10.05 | **0.00657** |
|  | Interaction | 2 | 4.79 | 0.09109 |
| Small and large | Age group | 1 | 2.28 | 0.13132 |
|  | Year | 2 | 12.49 | **0.00194** |
|  | Interaction | 2 | 6.14 | 0.04648 |
| Large | Age group | 1 | 8.06 | **0.00452** |
|  | Year | 2 | 17.14 | **0.00019** |
|  | Interaction | 2 | 7.61 | 0.02224 |
| Habitat class |  |  |  |  |
| Terrestrial | Age group | 1 | 2.96 | 0.08517 |
|  | Year | 2 | 3.75 | 0.15330 |
|  | Interaction | 2 | 4.86 | 0.08808 |
| Terrestrial and | Age group | 1 | 2.64 | 0.10432 |
| aquatic | Year | 2 | 20.87 | **0.00003** |
|  | Interaction | 2 | 6.57 | 0.03741 |
| Aquatic | Age group | 1 | 8.07 | 0.00449 |
|  | Year | 2 | 24.94 | **<0.00001** |
|  | Interaction | 2 | 8.26 | 0.01609 |

**Table A6** Dominance of the small mammal community and the number of small mammal catches per year in the Kis-Balaton marshland (Hungary). Y1 – 2014. Y2 – 2017. Y3 – 2020. MNA – minimum number alive (MNA) per 100 trap nights obtained by mark-recapture technique.

| Species | Y1 | Y2 | Y3 |
| --- | --- | --- | --- |
| *Apodemus sylvaticus* | 6.5 | 0 | 14.3 |
| *Apodemus flavicollis* | 0.6 | 0 | 0 |
| *Apodemus agrarius* | 45.2 | 62.5 | 10.7 |
| *Apodemus microps* | 1.9 | 0 | 0 |
| *Micromys minutus* | 0.6 | 0 | 0 |
| *Clethrionomys glareolus* | 18.7 | 12.5 | 32.1 |
| *Microtus subterraneus* | 2.6 | 0 | 3.6 |
| *Microtus arvalis* | 0.6 | 2.5 | 0 |
| *Microtus agrestis* | 8.4 | 5.0 | 17.9 |
| *Arvicola ambhibius* | 0 | 0 | 0 |
| *Crocidura leucodon* | 1.9 | 0 | 7.1 |
| *Sorex araneus* | 5.8 | 7.5 | 7.1 |
| *Sorex minutus* | 5.2 | 10.0 | 7.1 |
| *Neomys fodiens* | 1.3 | 0 | 0 |
| *Neomys anomalus* | 0.6 | 0 | 0 |
| MNA per 100 trap nights | 15.5 | 4.0 | 2.8 |


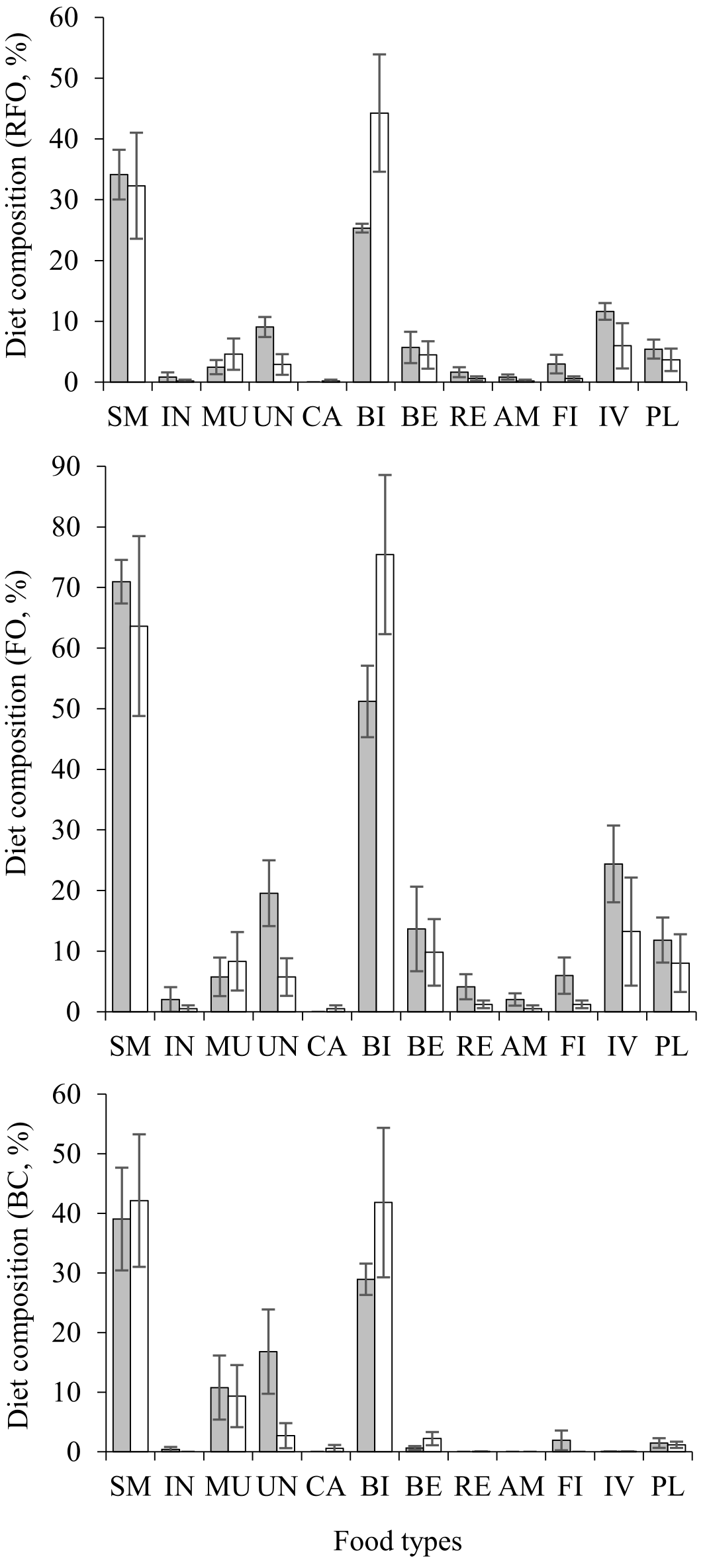


**Figure A1** Percentage relative frequency of occurrence (above), frequency of occurrence (in the middle) and estimated biomass (below) of main food categories in faecal samples of adult foxes (grey bars) and cubs (white bars) in the Kis-Balaton marshland (Hungary). Food types: SM – small rodents, IN – insectivores, MU – muskrat, UN – wild ungulates, CA – carnivores, BI – birds, BE – bird eggs, RE – reptiles, AM – amphibians, FI – fish, IV – invertebrates and PL – plants. Error bars represent the standard error of the mean.
